# Phosphorylation-Dependent Charge Transport in Biomolecular Junctions of Major Histocompatibility Complex Phosphopeptides

**DOI:** 10.1021/acs.jpcb.5c07407

**Published:** 2026-04-07

**Authors:** William Thompson, Jennifer Burrows, Reihaneh Safavi-Sohi, Mehrdad Rostami Osanloo, Hatef Sadeghi, Amanda Morgenstern, Ali Akbar Ashkarran

**Affiliations:** † Department of Physics and Energy Science, 14676 University of Colorado Colorado Springs, Colorado Springs, Colorado 80918, United States; ‡ BioFrontiers Center, 14676 University of Colorado Colorado Springs, Colorado Springs, Colorado 80918, United States; § Department of Chemistry and Biochemistry, 14676 University of Colorado Colorado Springs, Colorado Springs, Colorado 80918, United States; ∥ Department of Chemistry and Biochemistry, 5971 Seton Hall University, South Orange, New Jersey 07079, United States; ⊥ Department of Physics, the University of Texas at Dallas, Richardson, Texas 75080, United States; # Quantum Device Modelling Group, School of Engineering, 2707 University of Warwick, CV4 7AL Coventry, U.K.

## Abstract

This study investigates the hypothesis that phosphorylation states of major histocompatibility complex (MHC) proteins are correlated with their charge transport variations. We measured the rates of charge transport across self-assembled monolayers (SAMs) of MHC phosphopeptides containing phosphate groups at different sites supported on gold, with a Eutectic Gallium–Indium (EGaIn) top electrode. Measurements of the tunneling current densities across Au^TS^/MHC//Ga_2_O_3_/EGaIn molecular junctions show that the charge transport rates strongly depend on phosphorylation location. Surface characterization by AFM together with XPS and UPS confirms the formation of peptide SAMs and reveals variations in interfacial electronic structure. The presence of a phosphate group introduces localized dipoles that modify the tunneling barrier and interfacial energetics. Density functional theory (DFT) calculations suggest that phosphopeptides with increased charge transport rates have stronger dipole moments, which correspond to enhanced conductance due to a reduced energy barrier for electron transport. UPS measurements also indicate that phosphopeptides with increased charge transport have more negative HOMO energies. Our findings suggest the critical role of phosphorylation states in altering the fundamental charge transport characteristics across proteins.

## Graphical abstract

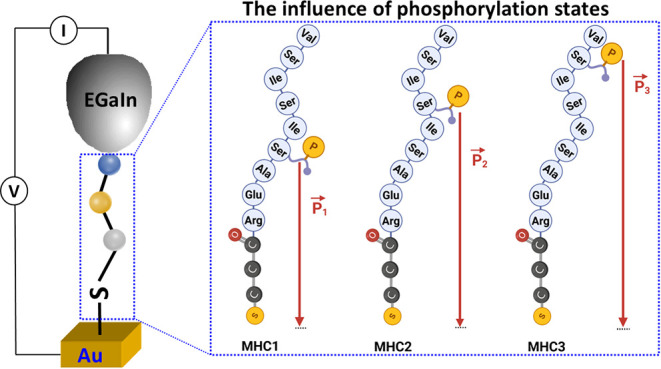

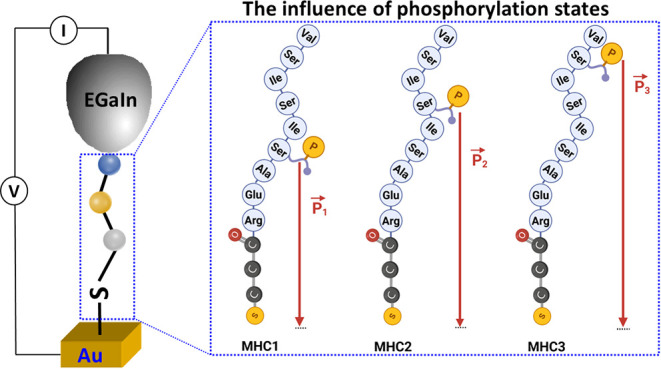

## 1 Introduction

Understanding charge transport across proteins is critical for several reasons, ranging from fundamental biological processes in many living organisms to biosensors and bioelectronic applications.^1–5^ Electron transport through biomolecules underpins a variety of essential biological processes, including respiration, photosynthesis, and enzymatic catalysis. By investigating how electron flow occurs across proteins and peptides, we can gain insight into the mechanisms of charge transfer in biological systems, which may reveal new strategies for modulating these processes for therapeutic purposes.^6–9^ In this regard, peptides and proteins offer an exciting platform for developing novel devices, owing to their highly tunable electronic properties, structural versatility, and biocompatibility.^1,6,10–12^ Charge transport depends on several parameters, including the sequence of amino acids forming the protein and conformational changes, and, therefore, provides versatile tools for regulating the charge transport within (bio) molecular electronic devices through chemical control.^13–15^

Among various proteins, major histocompatibility complex (MHC) proteins are of crucial importance in many biological systems as they play a fundamental role in various cellular processes (e.g., the body’s immune responses).^16–18^ Limited knowledge exists regarding charge transport through MHC proteins and, more specifically, about tunneling through molecules with various phosphorylation states.^2,19^ The question this study addresses is whether phosphorylation of proteins is associated with fundamental charge transport characteristics of proteins using MHC phosphopeptides (as a proof-of-concept model).

We studied the relative rates of electron transport by measuring current density through self-assembled monolayers (SAMs) of MHC phosphopeptides on gold thin films. The sequences of the peptides studied are [3Mpa]­RQASISISV (as MHC control), [3Mpa]­RQA­[pS]­ISISV (MHC1), [3Mpa]­RQASI­[pS]­ISV (MHC2), and [3Mpa]­RQASISI­[pS]V (MHC3) as representatives of phosphorylated peptides with various phosphorylation states (pS denotes phosphorylated serine residue). Note that three of these peptides contained phosphate groups at different serine residues, and one peptide had no phosphate group (as a control). 3-mercaptopropanoic acid (3Mpa) was used to covalently bond the phosphopeptides on the surface of the bottom gold electrodes, and using eutectic gallium–indium (EGaIn) alloy as the top electrode, we measured the charge transport rates across various MHC phosphopeptides. We also used density functional theory (DFT) simulations in an attempt to elucidate the origin of different charge transport rates across phosphopeptides with various phosphorylation states. We hypothesized that phosphorylation (i.e., addition of phosphate groups to a specific amino acid residue) of proteins significantly changes the electronic and charge transport properties of proteins.

These model sequences were selected based on previous studies that reveal phosphorylated peptides emerged as tumor-associated antigens presented by MHC molecules and identified by T cells, making them promising candidates for cancer immunotherapy.^16–18^ It was also reported that these antigens are attractive therapeutic targets, as RQApSlSISV is dysregulated in several solid tumors, implicated in the transformation process, and a target for chemotherapy.^20,21^ 3Mpa was selected based on our previous findings that short spacer molecules result in a lower overall tunneling barrier and less perturbation of electron transport mechanisms.^22,23^ Moreover, attaching linker molecules (i.e., 3Mpa) to the peptides during the synthesis process avoids extra formation steps and complexity in the deposition of SAMs of phosphopeptides.

## 2 Methods

### 2.1 Preparation of MHC Phosphopeptide Junctions

The overall workflow is shown in Figure 1. Three custom synthesized MHC phosphopeptides containing phosphate groups at various sites and one as control (i.e., without phosphate group), were ordered from JPT Peptide Technologies and prepared by immersion of a smooth template-stripped (TS) gold surface (Au^TS^) in 1.0 mg/mL 70% ethanolic solution of 3-mercaptopropanoic acid (3Mpa)-terminated molecules containing 10% Dimethyl sulfoxide (DMSO) overnight under a nitrogen atmosphere. Template-stripped Au surfaces (Au^TS^) were prepared based on reported protocols in the literature.^24–26^ After each immersion in EtOH, we gently rinsed the samples with 70% ethanol for 1 min (∼1 mL/min) to remove residue on the surface and dried the samples under a slow flow of nitrogen gas for 1 min.

**Figure 1:**
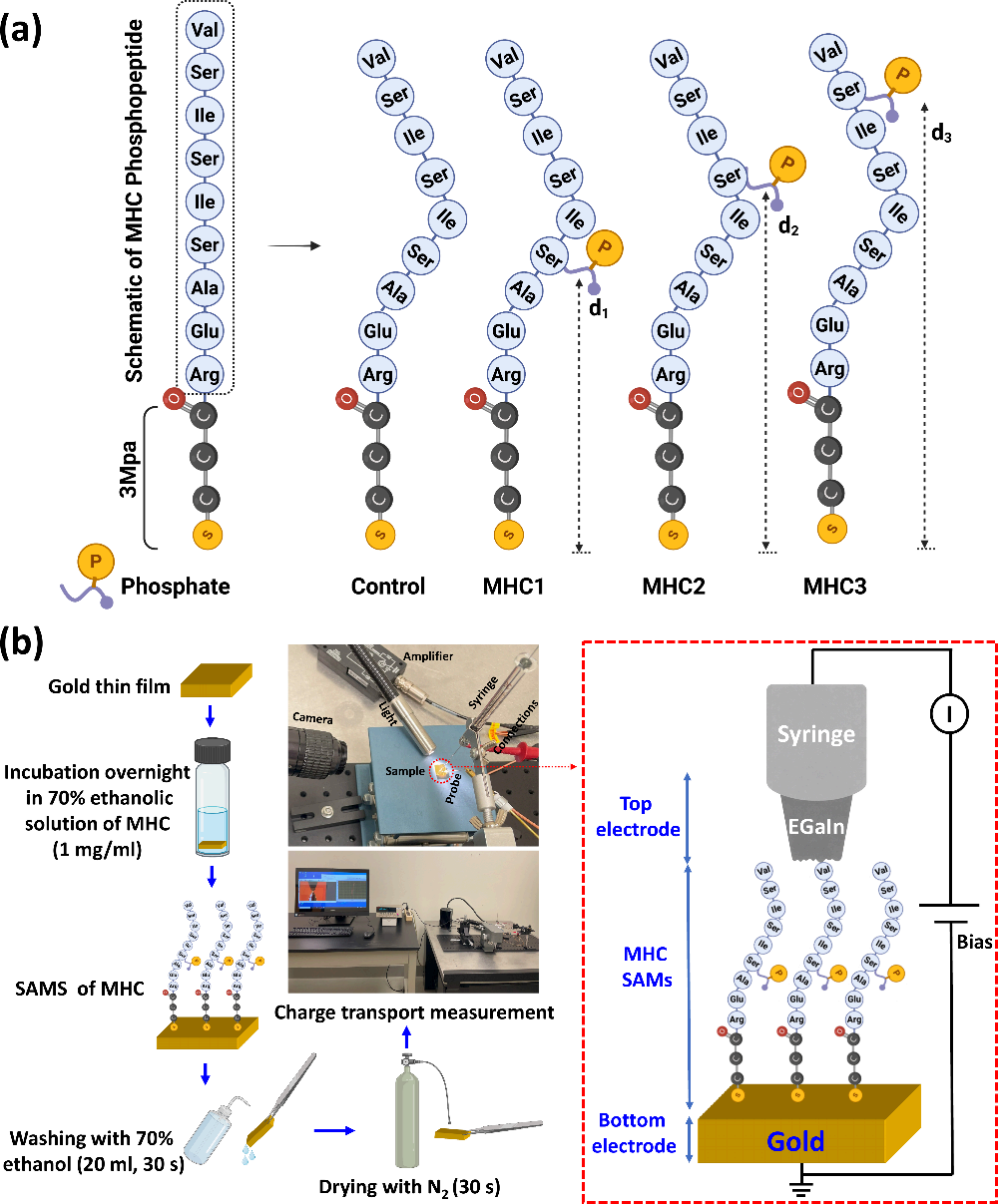
(a) Schematics show various phosphorylation states of MHC peptides upon the addition of phosphate group (denoted as “P”) on serine residues at different distances (d) from the sulfur atom and (b) schematic representation of the overall workflow for charge transport measurements across SAMs of Au^TS^/MHC//Ga_2_O_3_/EGaIn. The cartoons are meant to serve as schematics and are not to scale, and the SAMS of MHC peptides may not be as ordered as illustrated.

All current–voltage (*J*–*V*) measurements were performed on freshly prepared samples. After placing the samples on an antivibration table, we connected a grounded Au surface to the negative port of a source meter (6430 Sub-Femtoamp Remote SourceMeter, Keithley). A 10-μL Hamilton syringe containing eutectic indium–gallium (EGaIn, 75.5% Ga 24.5%, and superficial layer of GaOx) alloy, serving as a top electrode, was controlled by a micromanipulator and was connected to the port of the source meter. We formed an EGaIn tip of conical shape by extruding an EGaIn drop from the syringe on a clean Si wafer and bringing the EGaIn tip gently into contact with the samples (contact area ≈ 900 μm^2^).^27^ A voltage was subsequently applied to the EGaIn tip (a positive voltage corresponds to EGaIn oxidizing, and negative voltage corresponds to EGaIn reducing), and the current flowing across the junctions was measured (one trace from 0 to +0.5 V to −0.5 to 0 V).

### 2.2 Current Density Measurements

We created a “flattened” conical EGaIn tip by following a previously established protocol from the literature.^28,29^ After forming the flattened tip, we carefully lowered it onto the peptide-coated gold substrate to establish a gentle contact (Figure S1). The contact area, assumed to be circular, was measured by determining the diameter between the tip and the substrate using a calibrated CMOS camera. The diameter observed during each measurement was recorded and used as an input for current density calculations in the LabVIEW program. Voltages ranging from −0.5 to +0.5 V were applied in 50 mV increments, and the resulting current was measured using a Keithley 6430 Sub-Femtoamp SourceMeter.

### 2.3 Statistics

In all experiments, data were collected from three separate samples, consisting of independently prepared MHC phosphopeptides and control groups, and the average values were calculated. A standard 1–3–20 protocol was employed for collecting *J*–*V* data. More specifically, the 1–3–20 protocol indicates that a single initial *J*–*V* trace was first recorded to verify that the junction was not shorted. If the junction remained stable, three additional scans were collected at the same contact to further confirm stable behavior. After this verification step, 20 *J*–*V* traces were recorded, including both forward-bias and reverse-bias sweeps (*V* = 0 V → *V* = +0.5 V → *V* = −0.5 V → *V* = 0 V) from the same junction.^30^ For each individual experiment, between 7 and 12 junctions were analyzed per chip, with a total of 24 *J*(*V*) traces (forward and reverse biases) collected from each junction. This method resulted in the recording of few hundreds (i.e., between 300 and 600) *J* points for each applied voltage. All measured values of *J* (excluding short-circuited traces) at each voltage were included in the construction of log *J* histograms. These histograms were fitted with Gaussian curves to determine the log-mean values and log-standard deviations. Each individual log|*J*|−*V* plot represents the average of all trace-retrace pairs from a single experiment, and the final log|*J*|−*V* plots were calculated as the average of the three experiments

### 2.4 DFT Calculations

For density functional theory (DFT) simulations, the four peptides were built in the Amsterdam Modeling Suite (AMS) version 2023.104,^31^ starting with 3Mpa and adding the peptide backbone using the amino acid backbone tool. The amino acid side group tool was used to add onto the peptide chain. For the stretched conformations, each peptide used a fixed-atom constraint to prevent the peptide from folding, which was approximately 3.6 nm from the carbon atom adjacent to the thiol and valine’s carboxyl carbon atom. Partial geometry optimizations for stretched conformations utilized the hybrid PBE0 functional, an all-electron triple-ζ polarized (TZP) basis set,^32^ and scalar zero-order regular approximated Hamiltonian (ZORA) for relativistic effects.^33^ Default convergence values were used during the geometry optimization (gradient convergence of 1 × 10^–3^ Ha/Å, energy convergence of 1 × 10^–5^ Ha, and step convergence of 1 × 10^–2^ Å). Dipole moments were then calculated with the M06–2X functional and an all-electron TZP basis set.

To investigate other possible peptide conformations, a progressive level conformer search was performed starting with force field methods to generate around 2500 possible geometries for each peptide. Then density functional tight binding (DFTB) geometry optimizations were performed on the 60 conformations with the lowest force field energies. Finally, single point DFT calculations using the M06–2X functional were performed on the ten lowest energy DFTB conformations. From these results, both the lowest energy geometry and the lowest energy geometry that had an ideal binding orientation, meaning that the sulfur atom and terminal Val residue were positioned such that it would be possible to bind the gold surface and EGaIn tip, respectively, were retained for analysis. All peptides had a positive charge on Arg and a negative charge on the terminal Val from the removal of the H atom of the COOH group as has been done in other works.^34–36^ The control peptide thus had a neutral charge and each phosphorylated peptide had a minus one charge based on the relatively neutral pH during experiment.

## 3 Results and Discussion

We characterized the SAMs after formation to ensure that SAMs of the MHC peptides were successfully formed on the surface of gold thin films. Figures S2 and S3 of Supporting Information (SI) show the atomic force microscopy (AFM) images of SAMs of the three MHC phosphopeptides containing various phosphorylation states as well as the nonphosphorylated control and bare gold. AFM images shown in Figure S2a–d confirmed the formation of a homogeneous and relatively dense monolayer of MHC peptides with only minor pinholes and aggregates, which is consistent with previously published findings.^37–39^ AFM measurements were used primarily to assess the surface morphology and confirm the formation of peptide SAMs on gold surface. We note that AFM height variations may also arise from substrate roughness (Figure S3), defects, or partial coverage and therefore should not be interpreted as a direct measure of the electronic tunneling distance. It is noteworthy that the areas of the gold surface that are not fully covered by peptides are not expected to impact the final measurements or the charge transport properties. In fact, current density measurements in areas not fully covered by peptides result in short circuits due to the absence of molecules, and these regions are excluded from the final data. The SAMs of MHC phosphopeptides exhibited similar morphology and topography with each other and the control, as the peptide sequences are identical, with differences only in their phosphorylation sites. Although MHC phosphopeptides are covalently attached to the gold surface via 3Mpa, ensuring specific binding at one end, we acknowledge that there are no known specific binding sites on the MHC side interacting with the EGaIn tip. However, the relative uniformity of the SAMs, confirmed by AFM, suggests that the junctions likely contain multiple peptides aligned in parallel with a variable molecular arrangement and a variable height profile of a few nm (Figure S2). These results suggest that while some peptides may be stretched near their full length, other individual peptides may be in folded conformations as we discuss in more detail below. Therefore, we emphasize that the measurements represent the average charge transport properties across the junction, which may not always correspond to the full molecular length.

To investigate the chemical composition and electronic structure of the peptide-modified gold surfaces, X-ray photoelectron spectroscopy (XPS) and ultraviolet photoelectron spectroscopy (UPS) measurements were performed on the Au/SAM interfaces. The XPS analysis (Figures S4–S7) confirm the presence of the expected elements associated with the peptide monolayers, including C, N, O, and S signals, in addition to the Au signal from the substrate. The presence of sulfur is consistent with the thiol anchoring group used for SAM formation on the gold surface. In one of the phosphorylated peptides (MHC3), a detectable P signal (∼1.0 atom %) was observed, confirming the presence of the phosphate group in the sequence, while phosphorus was below the detection limit for the nonphosphorylated and MHC1 and MHC2 variants (Table S1). These results confirm successful formation of the peptide monolayers on the gold surface and demonstrate chemical differences associated with phosphorylation. Moreover, high-resolution spectra of the O 1s, N 1s, C 1s, S 2p, and Au 4f regions from XPS further support the formation of peptide monolayers on the gold substrate (Figures S8–S11).

The thickness of the peptide SAMs was estimated (Table S2) from attenuation of the Au 4f XPS signal using the exponential attenuation model, where the normalized inelastic mean free path is given by λ_n_ = λ_d_/ρ, and the overlayer thickness is determined from the intensity ratio of the Au signal for the bare substrate (*I*_0_) and the SAM-covered surface (*I*).^40^ Using a commonly adopted protein density of 1.35 g/cm^–3^,^41–43^ the calculated thickness values are approximately 1.2–1.3 nm for MHC control, MHC1, and MHC2, and ∼2.3 nm for MHC3. These values are smaller than the apparent heights obtained by AFM, which can include contributions from surface roughness and tip–sample convolution. In addition, the thickness obtained from XPS depends on the assumed density of the organic layer. Since peptide monolayers may have lower effective density than bulk proteins (i.e., 1.35 g/cm^–3^) due to incomplete packing or residual hydration, assuming a lower density increases the estimated thickness by ∼20 to 25%, while still remaining below the fully extended molecular length. In this regard, these results are consistent with tilted or partially disordered peptide conformations on the gold surface.^41^

UPS was used to probe the electronic structure of the Au/SAM interface (Figure S12). The work function values obtained from the secondary electron cutoff region range from 4.35 to 4.46 eV for the peptide-modified surfaces variants (Table S3). Compared with the typical work function of clean gold (∼5.1 eV), these values indicate the possible formation of an interfacial dipole associated with adsorption of the peptide monolayers. Small variations in work function are observed among the peptide variants, suggesting that the position of the phosphorylated serine residue influences the interfacial electrostatic environment and vacuum-level alignment. The HOMO onset energies obtained from UPS are in the range of 2.88–3.17 eV (binding energy), with MHC3 showing a slightly deeper HOMO level compared with the other peptides. These results indicate modest changes in the electronic structure of the SAM-modified surfaces associated with phosphorylation. Although UPS provides information on the electronic structure and vacuum-level shifts at the interface, the dominant transport orbital in molecular tunneling junctions cannot be determined solely from UPS measurements. These UPS results are also consistent with prior reports showing that interfacial dipoles within SAMs can strongly modulate work function, orbital alignment, and charge transport characteristics in metal/molecule junctions.^44,45^

Figure 2 demonstrates the *J*–*V* characteristics of SAMs of control and MHC peptides with various phosphorylation states on gold substrates and the corresponding log|*J*| histograms at +0.5 V (depicted next to each log|*J*|*–V* curve). The measurements for each sample were performed at several (7–12) random spots on three distinct prepared fresh chips of SAMs of MHC peptides and the corresponding average charge transport rates (i.e., *J*–*V* curves) were calculated and presented in Figure 3. More details on junction statistics, including the total number of junctions attempted, the number of shorted, and the corresponding yield of working junctions (i.e., the ratio of nonshorting junctions to all measured junctions) are presented in Table S4.

**Figure 2:**
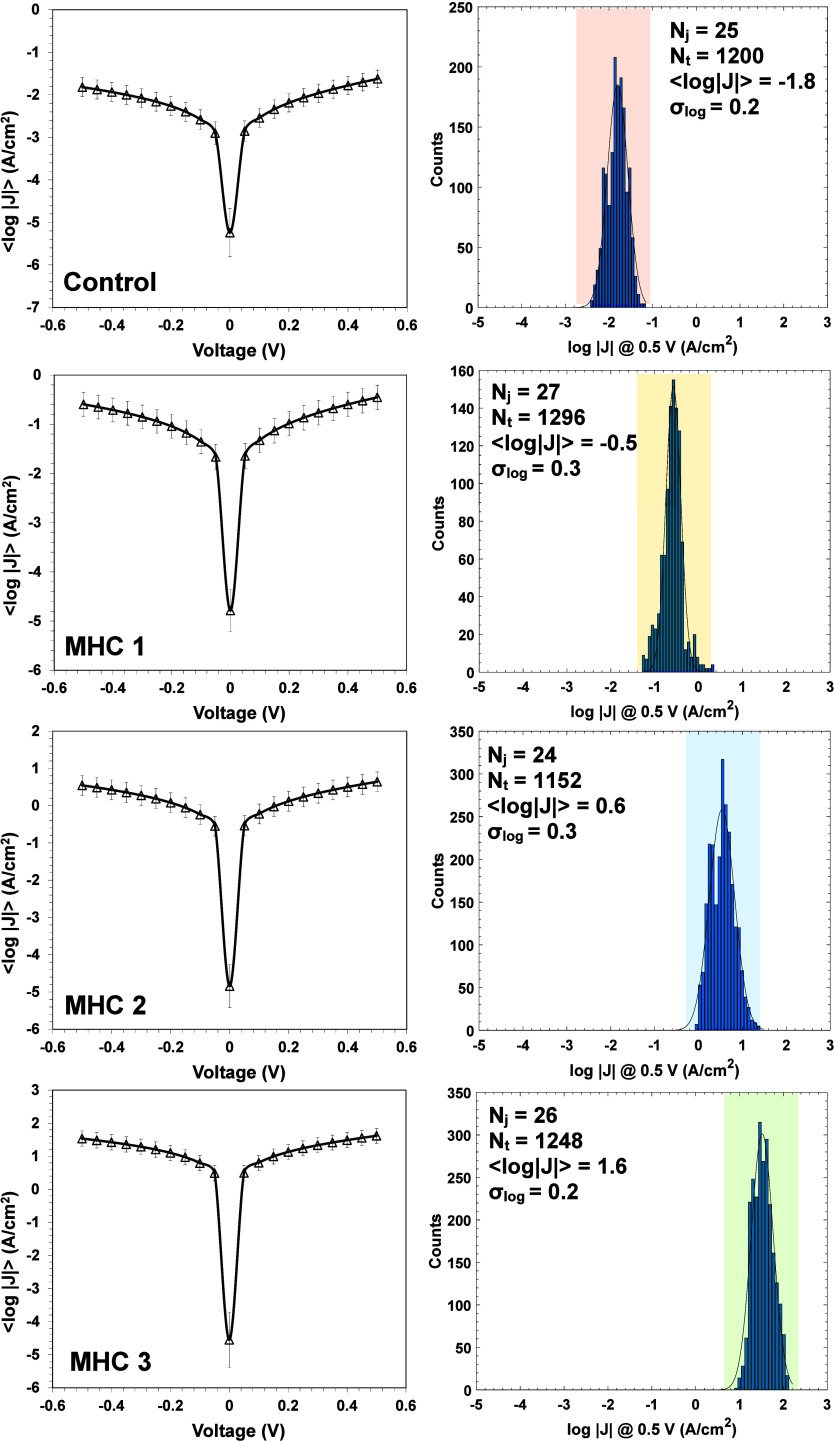
Current density (log|*J*|) plots versus applied voltage for Au^TS^/MHC//Ga_2_O_3_/EGaIn junctions with various phosphorylation states (i.e., MHC1, MHC2, MHC3, and nonphosphorylated sample as a control) and the corresponding histograms of current densities (at +0.5 V). (⟨log|*J*|⟩ is the population mean of log|*J*|, *N*_j_ is the total number of nonshorting junctions on three distinct measured chips, and *N*_t_ is the total number of traces performed in three distinct chips).

**Figure 3:**
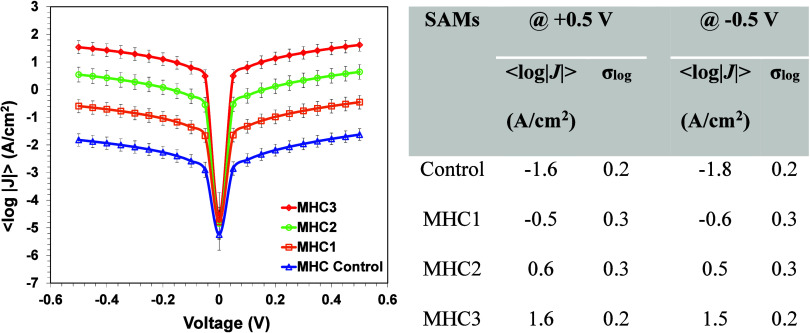
Electron tunnelling rate variations across SAMs of Au^TS^/MHC//Ga_2_O_3_/EGaIn containing various phosphorylation states and the corresponding average current density (log|*J*|) values (inset table) for SAMs of control and MHC peptides containing various phosphorylation states.

These results reveal that phosphorylation of the peptides significantly changes the electron transport across SAMs of MHC phosphopeptides. In fact, adding a phosphate group to the first serine residue (i.e., [3Mpa]­RQA­[pS]­ISISV) of the control sample (i.e., [3Mpa]­RQASISISV; SAMs of nonphosphorylated peptide), increases current by more than one-order of magnitude (see control and MHC1 in Figure 2). Moreover, by moving the phosphate group along the length of the peptide (i.e., adding a phosphate group to the second or third serine residues) further increases current by a few orders of magnitude. In other words, the location of the phosphorylation site of the peptide critically affects its electron transport properties across the peptide.

It is reported that many proteins in eukaryotic cells may have up to an average length of ∼400 amino acids.^46,47^ Approximately 17% of these amino acids are Ser (8.5%), Thr (5.7%), and Tyr (3.0%), which are potential sites for phosphorylation and among the mentioned amino acids, serine residues have a high chance of being phosphorylated.^48,49^ That is why, in the initial design of the peptides, we used serine residues as the potential site for the phosphorylated model peptide. It is found that the charge transport characteristics strongly depend on the phosphorylation states of the MHC phosphopeptides. By varying the phosphorylation state of the serine residues occurring at every other reside position in the phosphopeptides, current increase by approximately linearly 1 order of magnitude (Figure 3). In fact, phosphorylation changes the electronic structure of the peptides, inducing changes in rates of charge transport across the whole molecular junction.^6^ The negative charge of the phosphate group also results in a different dipole moment that varies by position of the phosphate group and/or phosphorylation states (Figure 4).^50,51^

**Figure 4:**
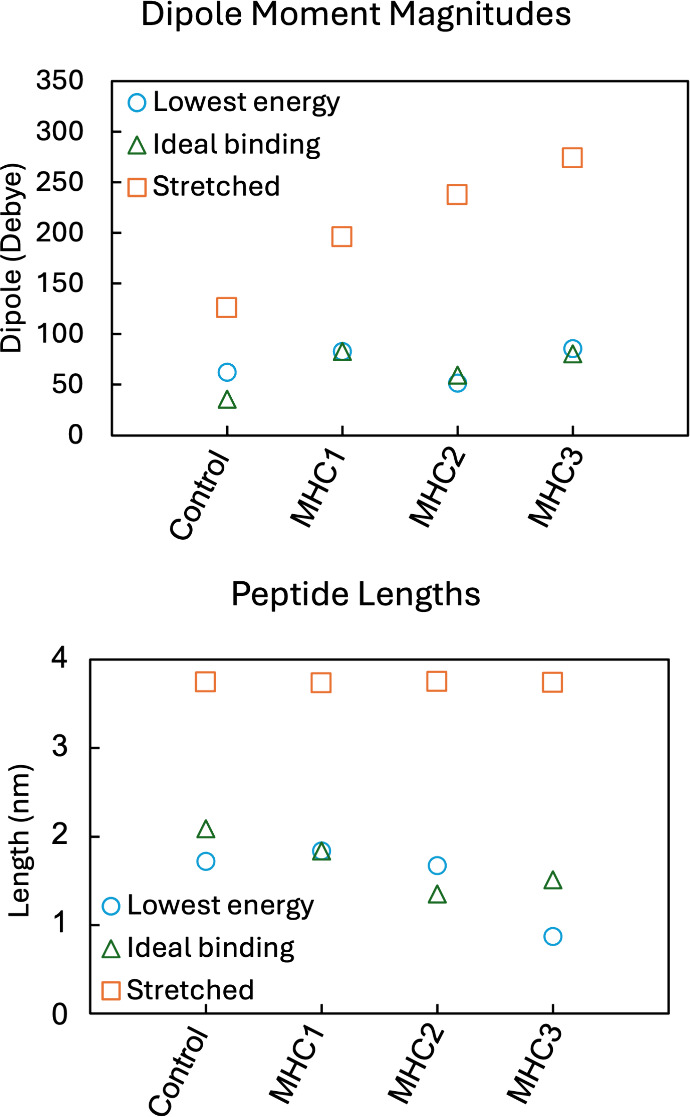
Dur MHC peptides studied. Three conformations are included for each peptide with data for the dipole moment magnitudes and length of the peptides measuring from the 3Mpa sulfur atom to the terminal Val oxygen atom included for stretched and ideal binding conformations. For lowest energy conformations, the lengths are measured from the sulfur atom to varying oxygen atoms depending on the geometry (see Figure S13).

Also, the observed work-function shifts and HOMO variations in UPS analysis indicates that phosphorylation possibly modifies the interfacial dipole and electronic structure of the peptide monolayers, which may contribute to the differences observed in charge transport across the molecular junctions. It is noteworthy that small current asymmetry at ±0.5 V can arise from interfacial effects and electrode asymmetry, even in junctions that do not function as molecular diodes (e.g., MHC). In fact, molecular rectifiers in large-area EGaIn/SAM junctions that are typically described as “rectifying” commonly exhibit rectification ratios (RR) of one to 3 orders of magnitude (e.g., RR ∼ 10^1^–10^3^) over an appropriate voltage window.

According to the Landauer formula, the conductance G in coherent tunneling depends exponentially on the molecular length *d*:^52,53^

$$G\propto e^{-\beta d}$$ 1

where β is the decay constant (typically 0.7–1.2 Å^–1^ for peptides), and *d* is the tunneling distance.^8^ Because the peptide sequences have similar backbone lengths and comparable SAM thicknesses, extraction of a reliable tunneling decay constant β from the experimental data is not feasible. Instead, the assumed β value is used only for order-of-magnitude comparison with typical molecular junction systems. Considering the number of amino acids in the peptide (i.e., nine, excluding the 3Mpa linker) and assuming a mostly stretched conformation, we estimate a conductance of approximately 10^–13^ *G*_0_, resulting in a current of ∼10^–18^ A/molecule (assuming a nonapeptide with a length of *d* = 3 nm and a decay constant β = 1.0 Å^–1^).^54^ Given a bias voltage of 0.5 V, we have *I* = *G* × *V* ≈ 10^–18^ A/molecule. For a SAM with a typical cross-sectional area of 1 nm^2^ per molecule therefore, the upper limit of the current density would be *J* ≈ 10^–18^ A/molecule × 10^14^ molecules/cm^2^ ≈ 10^–4^ A/cm^2^.^8^ However, the experimentally observed current densities (Figure 3) are a few orders of magnitude higher than this theoretical estimate. This indicates that either a pure coherent tunneling model cannot fully account for the observed values or the peptides are not all in their mostly stretched conformations.^55^

Based on our experimental findings, we propose two possible alternative mechanisms including hopping transport and multistep tunneling. The hopping mechanism involves charge carriers jumping between localized states along the peptide chain, which becomes more possible when there is a significant energy difference between the molecular orbitals and the Fermi level of the electrodes, thereby reducing the probability of direct tunneling. In this case, the HOMO energies were experimentally determined to be between −2.88 and −3.17 eV while the Fermi level of the gold electrode is approximately −5.1 eV. This energy alignment suggests that for some peptides—particularly those with HOMO levels near the Fermi level—direct tunneling remains a viable pathway for charge transport. However, for others where the energy mismatch is more pronounced, the tunneling probability may drop remarkably. On the other hand, multistep tunneling refers to the fact that electrons cannot travel through the whole peptide in one shot and instead they tunnel through short parts and then hop between those regions. This is another possible mechanism in MHC phosphopeptides because of their flexible structure. These peptides are not rigid rods and therefore, can bend or form loops, which might bring parts of the molecule closer together and shorten the distance electrons need to travel.^56,57^ For example, in some systems like helicenes, it has been shown that folded shapes can actually improve electron transport because they help the molecular orbitals overlap better.^58,59^ In fact, it is possible that folded peptides could make it easier for charges to move by creating shorter or more efficient pathways, even though the overall molecule is still long. That may explain why our measured current densities are higher than what simple tunneling models predict.

Additionally, the dielectric environment of SAMs can influence charge transport by modifying the effective tunneling barriers and interfacial electrostatics within molecular junctions.^60^ Variations in molecular polarization and dielectric screening within the SAM may therefore affect the alignment of electronic states and the local electric field across the junction.^61^ Recent studies have shown that the dielectric response of molecular layers can impact the transport characteristics in large-area molecular junctions.^62,63^ Although a detailed analysis of dielectric effects is beyond the scope of the present work, we note that dielectric screening within peptide monolayers may contribute to the observed transport behavior and represents an interesting direction for future work.

To further explore the origin of the electron tunneling differences among MHC phosphopeptides containing various phosphorylation locations at the atomic level, we performed gas phase quantum mechanical calculations to determine the most energetically favorable peptide conformations and dipole moments. Calculations included individual peptides with 3Mpa but did not include the Au or EGaIn. Therfore, we recognize that these single molecule, gas phase calculations do not capture all the interactions involved in the SAMs and are used as an approximation of the true system.^64,65^

Geometries for the low energy and ideal binding conformations are shown in Figure S13. Possible peptide lengths and dipole moments were analyzed for stretched, low energy, and ideal binding geometries of all four MHC peptides as shown in Figure 4. Figure 5 shows the shapes of the HOMOs for the stretched geometries of each peptide (see SI for optimized atomic coordinates and Figure S13 for HOMOs of peptides in other conformations). While the addition of phosphate groups to different serine residues slightly affects the HOMO energy as found in UPS experiments, it likely does not drastically alter the localization of the HOMO as shown from DFT calculations. The conformation of each peptide also does not appear to have a large effect on orbital shape and localization. In all cases, the HOMO is localized on the negatively charged Val carboxylate from gas phase single peptide calculations.

**Figure 5:**
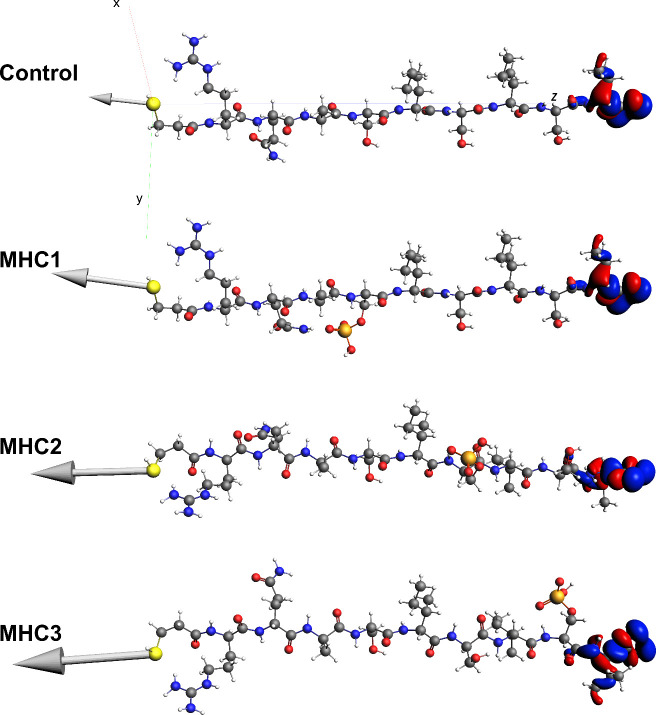
HOMOs and dipole vectors for control, MHC1, MHC2, and MHC3 in their stretched conformations, from top to bottom. The coordinate axis used for all peptides is shown for the control.

DFT calculations reveal that phosphorylation also affects the dipole moment of phosphopeptides. As the negatively charged phosphate group is moved further along the peptide from the 3Mpa linker, the dipole moment magnitude increases as shown in Figure 4. Most of this change occurs in the z-component of the dipole moment vector, which is parallel to each peptide for the stretched conformation. The lowest energy conformation of MHC3 is the only instance where the z-component of the dipole varies greatly from the overall dipole magnitude (see Table S5). For all conformations, the dipole moment magnitude generally increases for the peptides that have higher current densities from experiment, in line with increased dipole moments facilitating charge transfer through molecules. It is noteworthy that the calculated dipole moment magnitudes are relatively large because calculated dipole moments depend strongly on the choice of coordinate origin for charged molecules. In our calculations the origin was defined at the sulfur atom of the linker bound to the gold surface, while negatively charged phosphate and carboxylate groups are located farther along the peptide backbone, which increases the computed dipole magnitude. Molecular dipoles in SAMs are known to influence the local electrostatic environment and modify the metal work function and interfacial energy-level alignment. Such dipole-induced shifts can alter the effective tunneling barrier and therefore contribute to variations in charge transport observed across peptide-based molecular junctions.^66–68^

Peptide length is also related to conductance as shown in eq 1. We used a conformer search to investigate possible peptide lengths. For the stretched and ideal binding conformations, we defined peptide length as the distance from the 3Mpa sulfur atom to an oxygen atom on the terminal Val. For the lowest energy conformation, the terminal Val is not always in a position that would allow binding to the EGaIn tip. In these cases, we instead measured the length from the sulfur atom to another oxygen atom that would be geometrically capable of binding to the EGaIn tip (see Figure S13). For the stretched conformations, all peptides had similar lengths around 3.7 nm. For the folded conformations, the lengths of the peptides varied. However, Figure 4 shows that there is a general trend of decreasing peptide length as phosphate groups are added further along the peptide chain from MHC1 to MHC3. When comparing to experimental data, the SAM thicknesses were estimated as 1.2–1.3 nm for all phosphopeptide SAMs except MHC3 which had a thickness of 2.3 nm. The 1.2–1.3 nm thicknesses are similar to the low energy and ideal binding geometry lengths calculated. However, the 2.3 nm thickness measured for MHC3 is in between these two geometries and the stretched geometry length. Shorter peptide geometries should lead to increased charge transfer, which matches the general trends from DFT lengths calculated but is not in line with the experimental thickness of the MHC3 SAM. Further work is needed to verify the exact structures of the SAMs and understand interactions between and within individual peptides.

## 4 Conclusions

In summary, we show that the phosphorylation states of MHC proteins are linked to variations in their electron transport properties. We measured charge transport across SAMs of MHC phosphopeptides containing phosphate groups at different sites in molecular junctions and found that the charge transport rates are highly dependent on the phosphorylation status of the peptides. Surface characterization using AFM, XPS, and UPS confirms the formation of peptide SAMs and reveals variations in interfacial electronic structure associated with phosphorylation. The introduction of a phosphate group generates localized dipoles within the molecular layer, which can modify the tunneling barrier and interfacial energetics. Our findings suggest that incoherent transport mechanisms, such as hopping or multistep tunneling, may contribute to the observed current densities. Our results also reveal that the introduction of a phosphate group induces a localized electric dipole moment in the SAMs which alters the tunneling barrier, HOMO, and total dipole moment, leading to significant variations in charge transport rates. Our DFT calculations further indicate that phosphopeptides with elevated charge transport rates possess stronger dipole moments and may also have shorter peptide lengths. These results highlight the crucial influence of phosphorylation states in modifying the intrinsic charge transport properties of proteins.

## Supplementary Material



## Data Availability

All relevant data are available from the authors.

## Notes

Experimental and quantum mechanical calculations, details and additional supporting data, and the related discussion, including AFM, XPS, UPS of SAMs of peptides (PDF)
