# Supplementary material for: Phosphorylation-Dependent Charge Transport in Biomolecular Junctions of Major Histocompatibility Complex Phosphopeptides
Source: J Phys Chem B. 2026 Apr 7;130(15):4070–9. doi: 10.1021/acs.jpcb.5c07407 (PMC13093478; doi:10.1021/acs.jpcb.5c07407)
Supplement: Supplementary file 1 [file jp5c07407_si_001.pdf]

Supplementary Information

Phosphorylation-Dependent Charge Transport in Biomolecular Junctions of Major Histocompatibility Complex Phosphopeptides

William Thompson[1,2], Jennifer Burrows[3], Reihaneh Safavi Sohi[4], Mehrdad Rostami Osanloo[5], Hatef Sadeghi[6], Amanda Morgenstern[3]*, Ali Akbar Ashkarran[1,2]*

[1]Department of Physics and Energy Science, University of Colorado Colorado Springs, Colorado Springs, 80918, CO, USA

[2]BioFrontiers Center, University of Colorado Colorado Springs, Colorado Springs, 80918, CO, USA

[3]Department of Chemistry and Biochemistry, University of Colorado Colorado Springs, Colorado Springs, 80918, CO, USA

[4]Department of Chemistry and Biochemistry, Seton Hall University, South Orange, 07079, NJ, USA

[5]Department of Physics, the University of Texas at Dallas, Richardson, 75080, TX, USA

[6]Quantum Device Modelling Group, School of Engineering, University of Warwick, CV4 7AL Coventry, United Kingdom

*Corresponding authors (A.M, A.A.A): (amorgens@uccs.edu; aashkarr@uccs.edu)

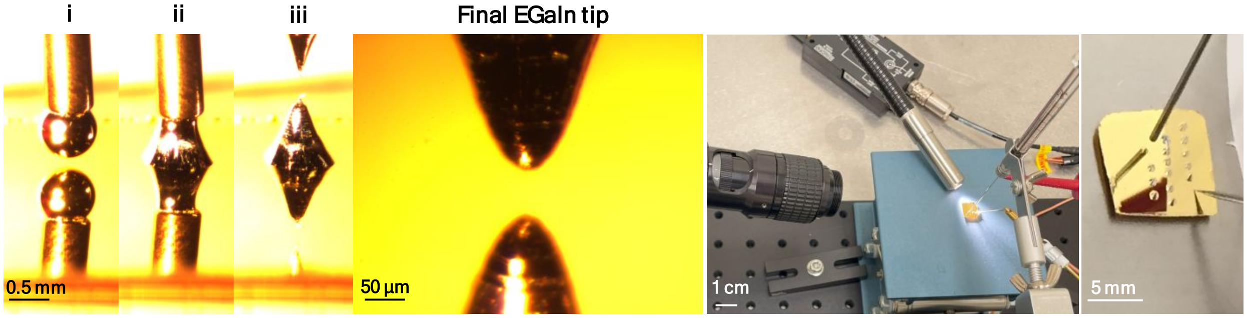

Figure S1: The images display the sequential formation process of the EGaIn top electrode tip (left to right) and a close view of the measurement setup and junction.

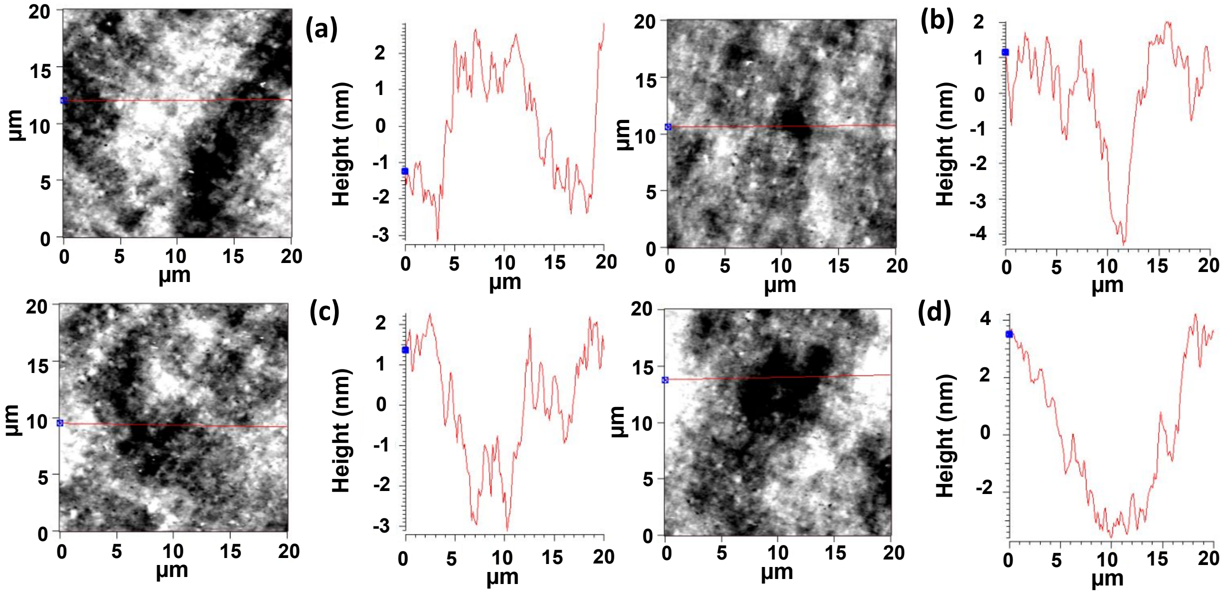

Figure S2: Formation of SAMs of MHC phosphopeptides on gold surface. AFM images and the corresponding height profiles of SAMs of (a) control, (b), MHC1 (c), MHC2, and (d) MHC3.

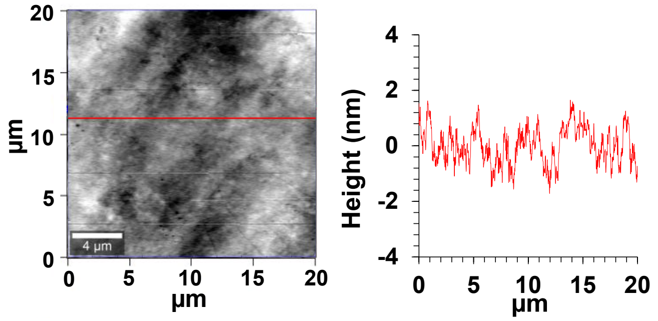

Figure S3: AFM image of bare gold surface and the corresponding height profile, as control.

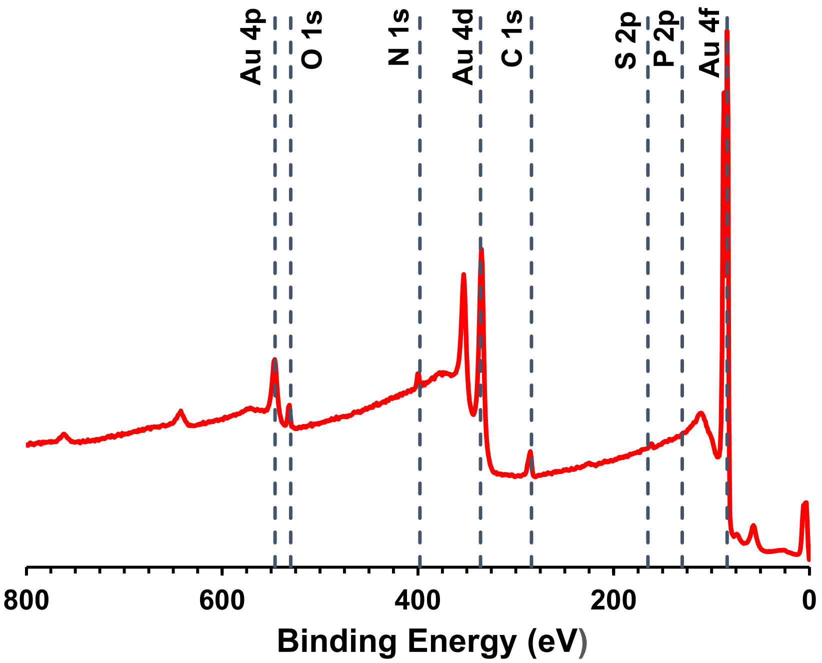

Figure S4: Full survey XPS scan of MHC – Control highlighting elemental peaks versus binding energy.

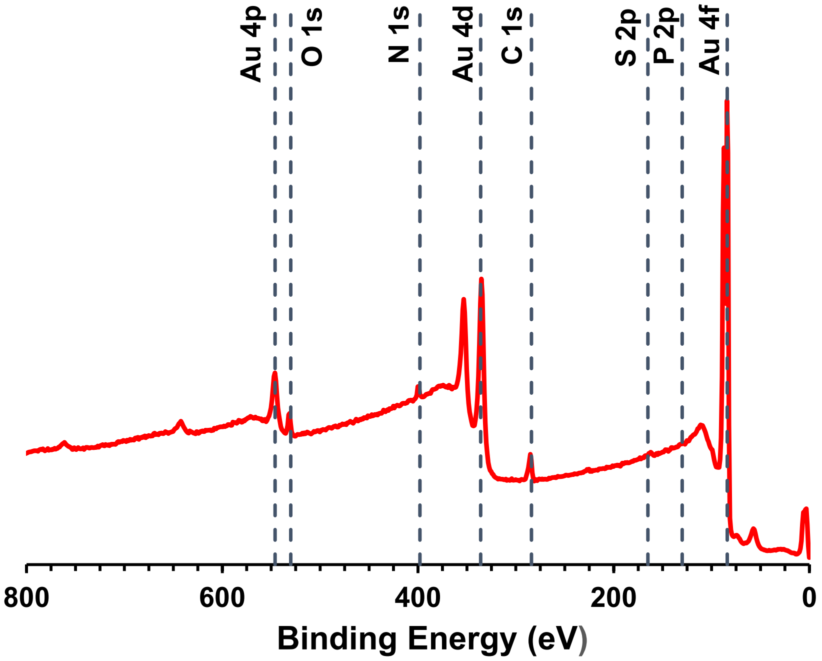

Figure S5: Full survey XPS scan of MHC1highlighting elemental peaks versus binding energy.

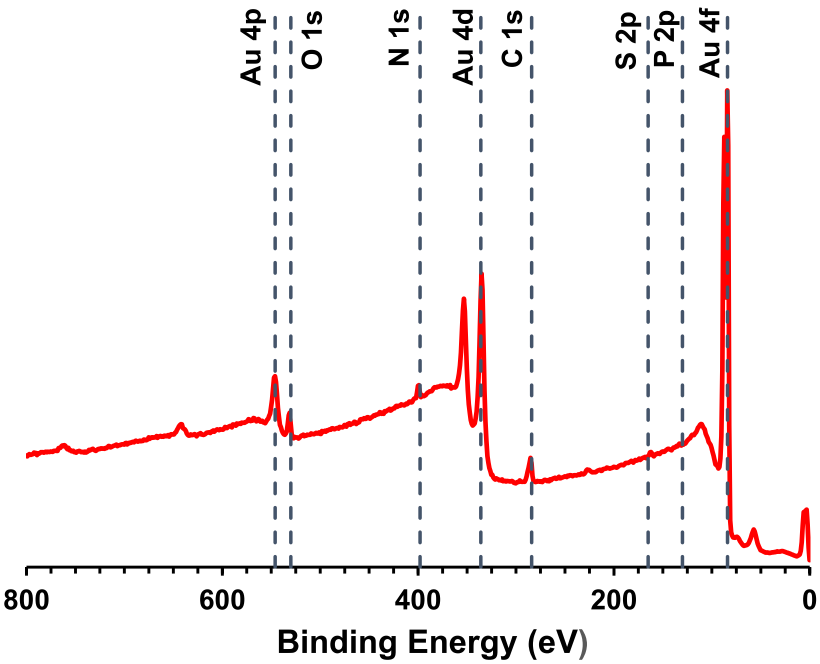

Figure S6: Full survey XPS scan of MHC2 highlighting elemental peaks versus binding energy.

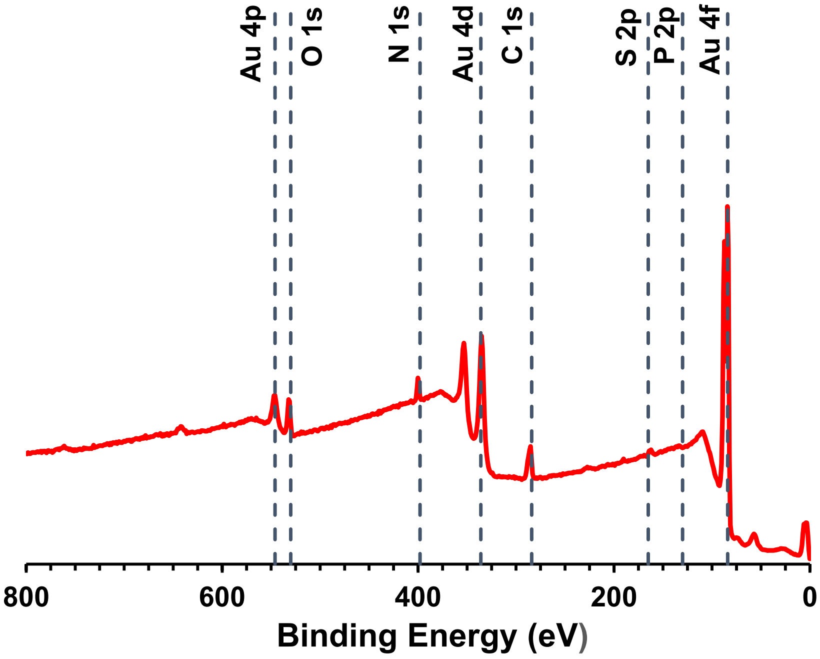

Figure S7: Full survey XPS scan of MHC3 highlighting elemental peaks versus binding energy.

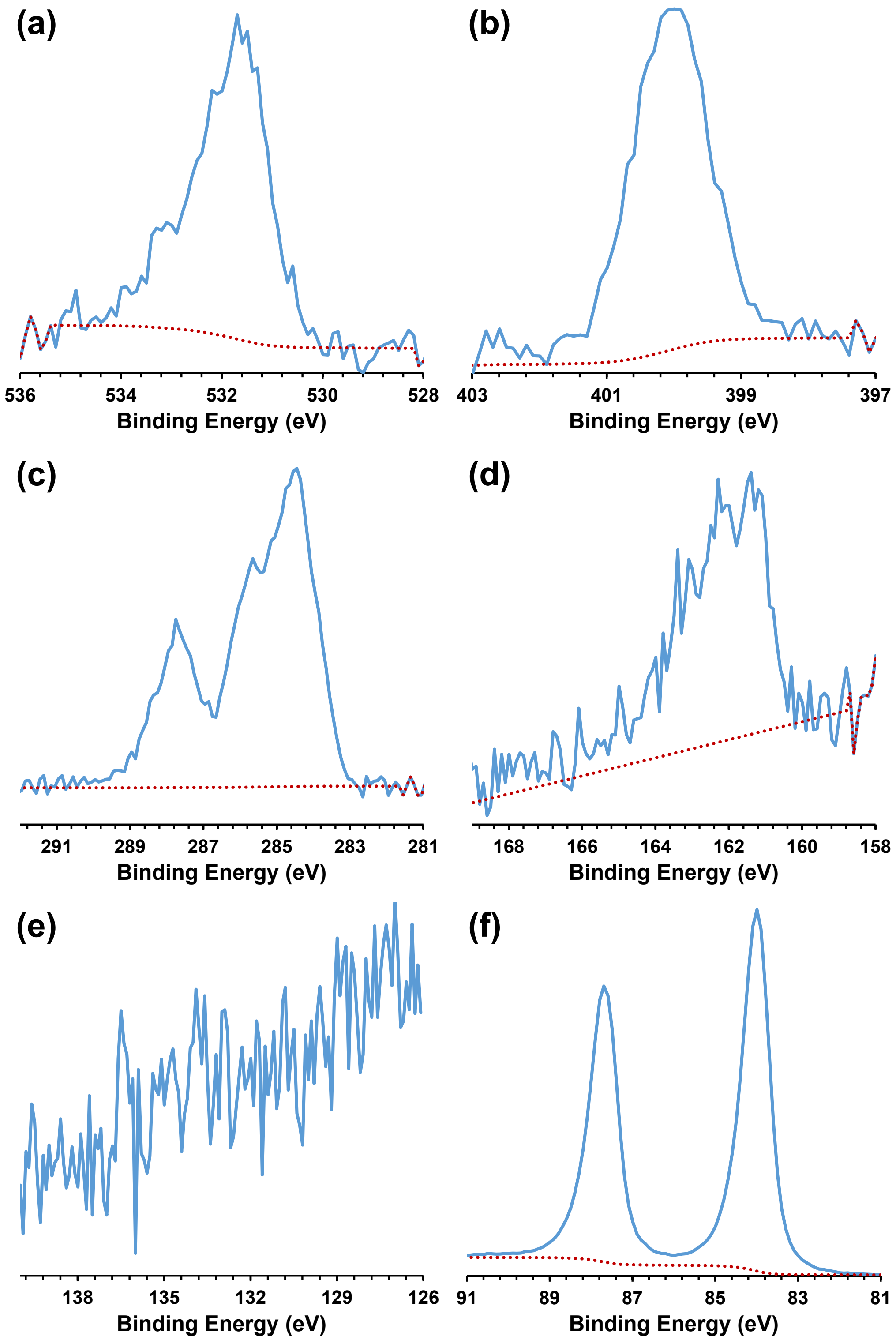

Figure S8: High resolution XPS scans of MHC – Control zoomed in on peaks versus binding energy for a) O 1s, b) N 1s, c) C 1s, d) S 2p, e) P 2p, f) Au 4f.

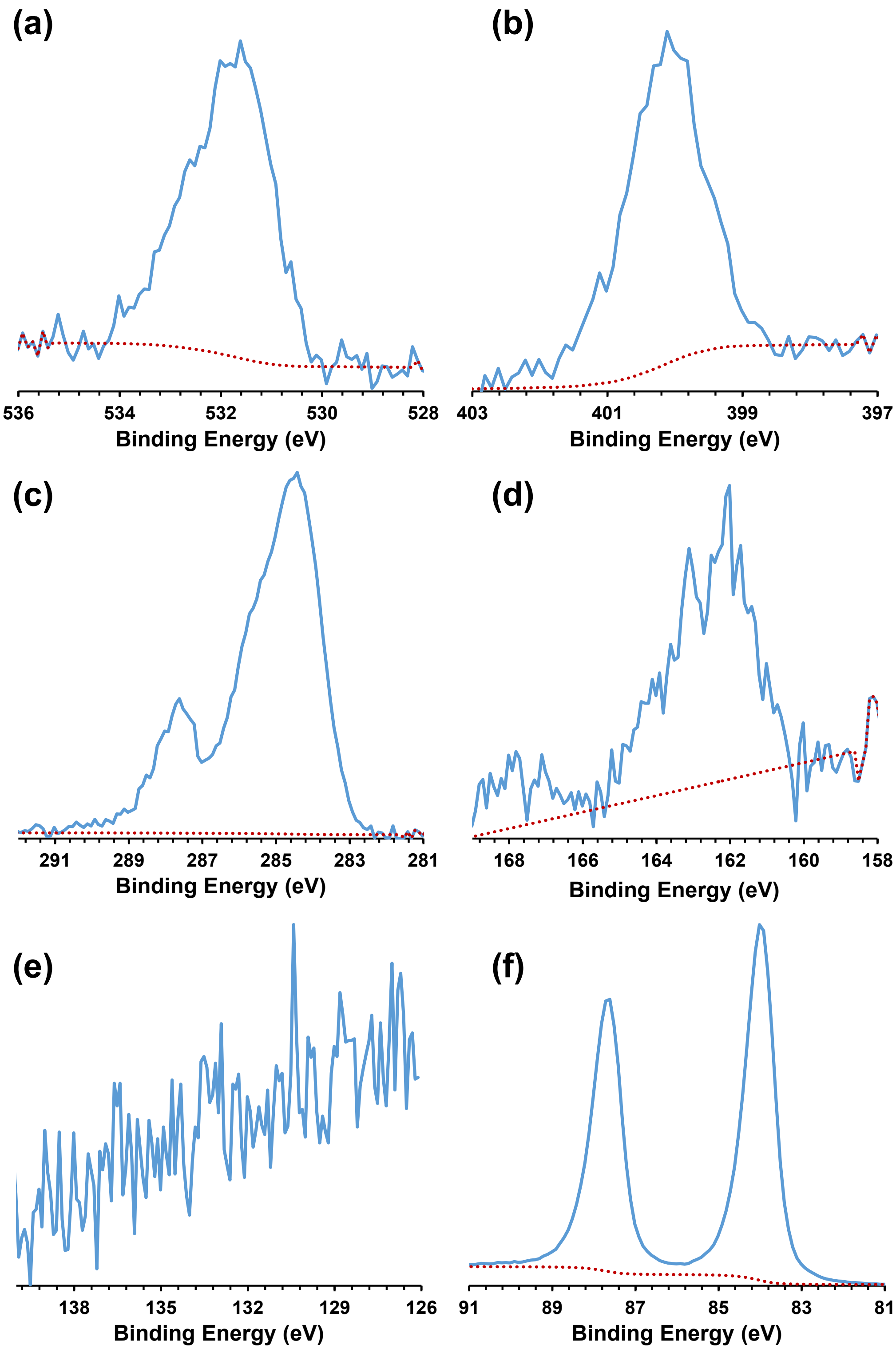

Figure S9: High resolution XPS scans of MHC1 in on peaks versus binding energy for a) O 1s, b) N 1s, c) C 1s, d) S 2p, e) P 2p, f) Au 4f.

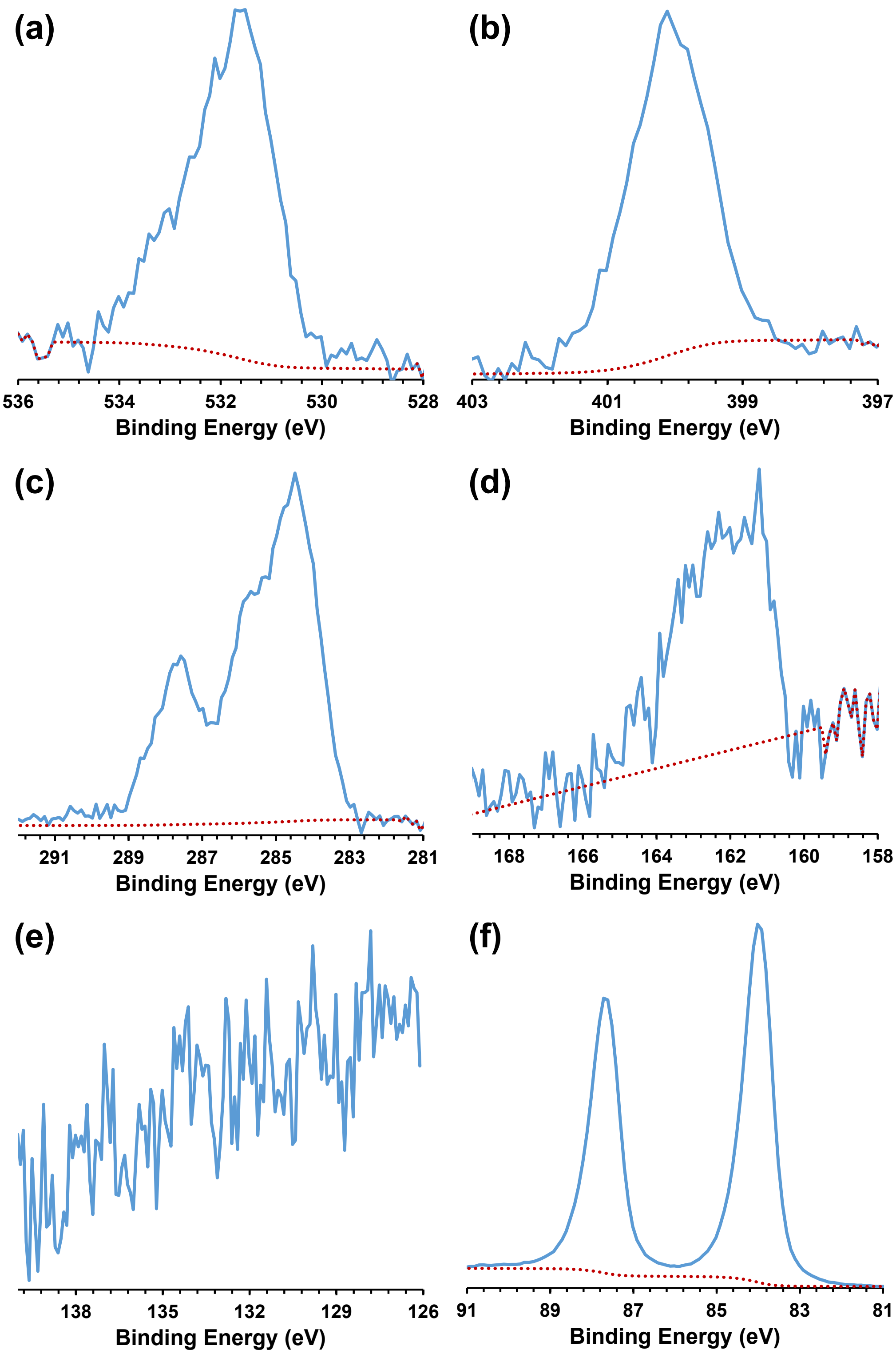

Figure S10: High resolution XPS scans of MHC2 in on peaks versus binding energy for a) O 1s, b) N 1s, c) C 1s, d) S 2p, e) P 2p, f) Au 4f.

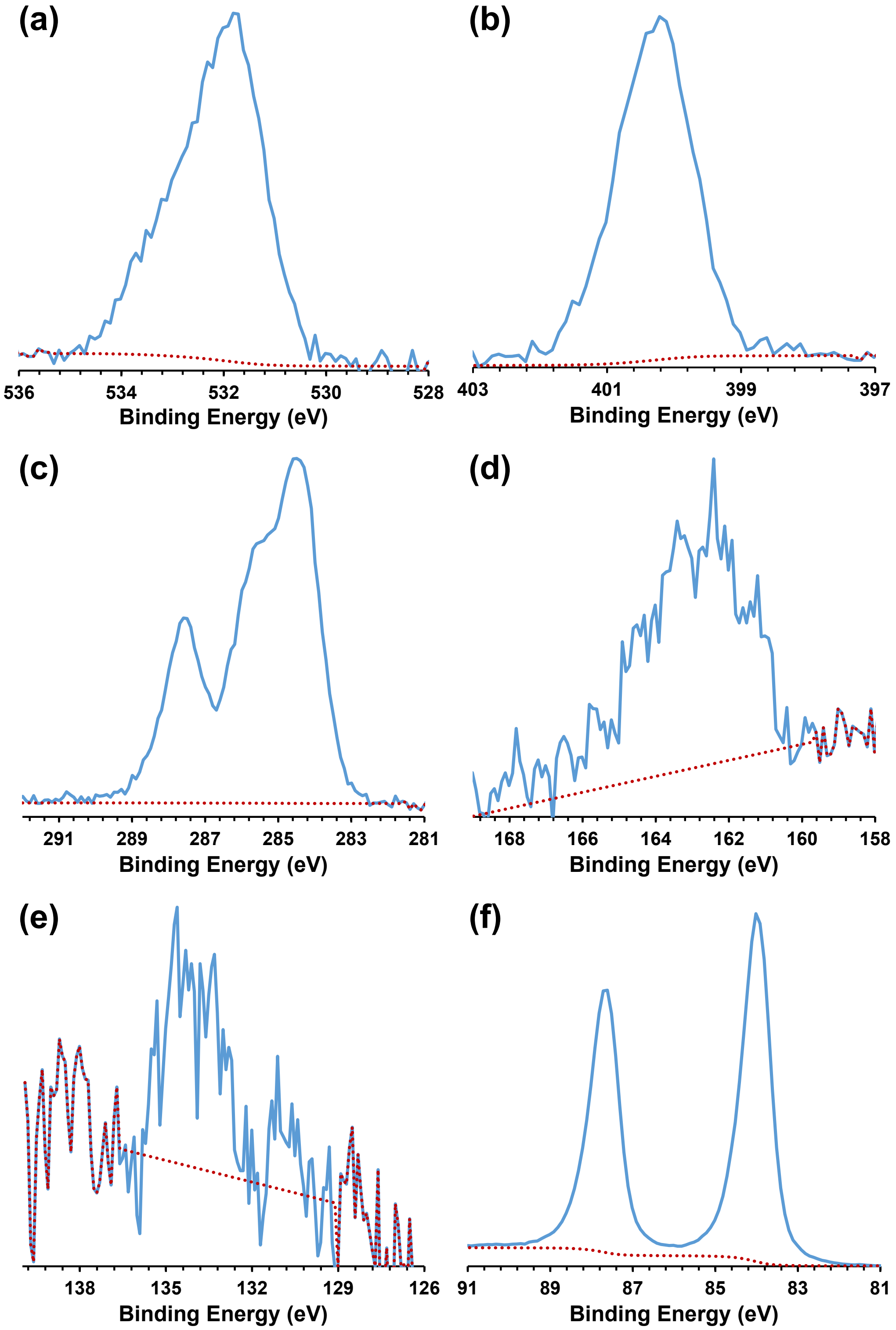

Figure S11: High resolution XPS scans of MHC3 in on peaks versus binding energy for a) O 1s, b) N 1s, c) C 1s, d) S 2p, e) P 2p, f) Au 4f.

Table S1: Elemental components reported as relative atomic percent for SAMS of MHC.

Sample	O	N	S	P	Au	C
MHC control	9.4	9.1	3.3	<LOD	42.3	36.0
MHC 1	9.8	7.5	3.4	<LOD	37.7	41.7
MHC 2	11.6	8.9	2.7	<LOD	37.6	39.2
MHC 3	14.2	11.8	3.5	1.0	22.9	46.7

Table S2: Overlayer thickness calculated from attenuation of Au 4f signal

Sample	Average Peptide density, ρ (g/cm^3)	$\lambda_d = (49/E^2 + 0.11E^{1/2})^b$ (mg/m^2)	$\lambda_n = \lambda_d/\rho$ (nm)	Au 4f intensity, I (cps)	Overlayer thickness $d = \lambda_n \ln(I_0/I)$ (nm)
MHC control	1.35	4.1	3.1	233645.7	1.3
MHC 1	1.35	4.1	3.1	226426.3	1.3
MHC 2	1.35	4.1	3.1	233826.2	1.2
MHC 3	1.35	4.1	3.1	167401.8	2.3

Table S3: UPS measurements showing work functions and HOMO for SAMS of MHC.

Sample	Work Function, ϕ (eV KE)	HOMO (eV BE)
MHC control	4.35	2.88
MHC 1	4.41	2.88
MHC 2	4.46	2.91
MHC 3	4.38	3.17

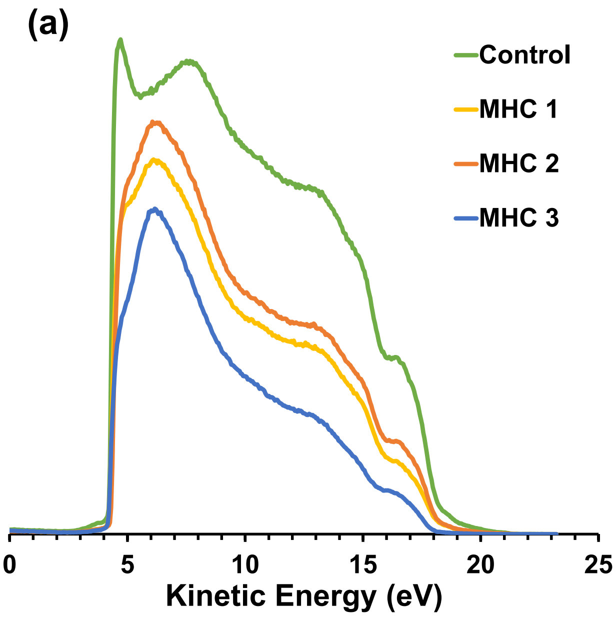

Figure S12: UPS Spectra of all MHC plotted together against a) kinetic energy to demonstrate work function determined by extrapolating the midpoint of the vertical secondary electron cut-off (SECO) to the KE and b) binding energy to demonstrate HOMO determined by extrapolating the valence electron drop to the BE.

Table S4: Summary of junction attempts, stability, and yields for Au^{TS}/MHC//Ga_2O_3/EGaIn junctions.

SAMs	Total junctions attempted	Stable (non-shorting)	Shorts	Junction yield (%)
**Control**	25	22	3	88
**MHC1**	27	19	8	70
**MHC2**	24	18	6	75
**MHC3**	26	21	5	80

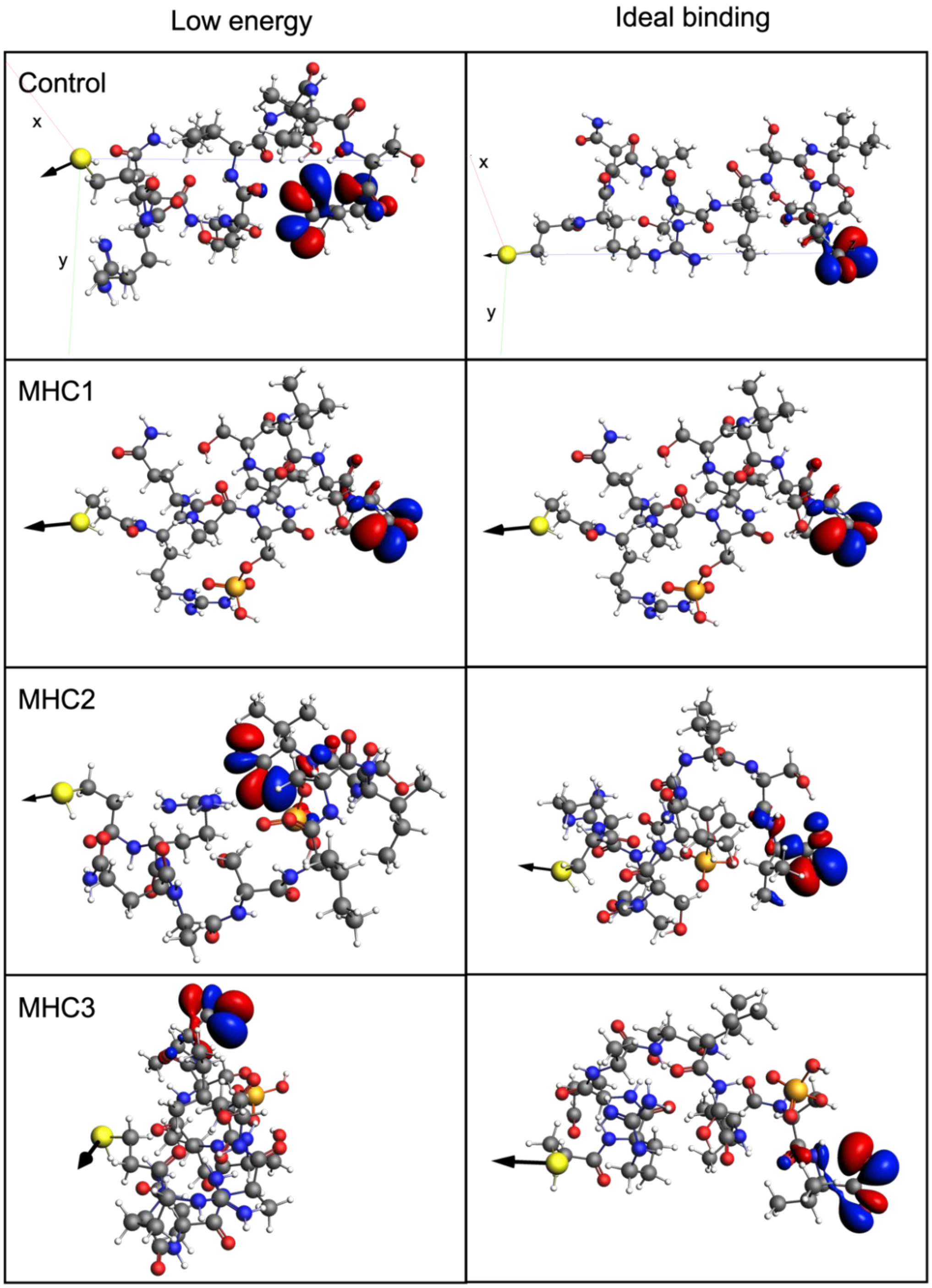

Figure S13: HOMOs for all four peptides tested in two different folded conformations. The left column shows the absolute lowest energy conformations, and the right column shows the lowest energy conformations found that also result in the thiol sulfur atom and Val carboxylate in positions that could ideally bind to the gold surface and EuGaIn tip, respectively, along the z-axis. The coordinate axes used are shown for the control molecules in the top row and are oriented identically for MHC1, MHC2, and MHC3. Dipole moments with relative magnitudes are shown in black vectors centered on each sulfur atom. Note that the low energy and ideal binding geometries are identical for MHC1.

Table S5: Dipole moment z-component and total magnitude for each phosphopeptide for all three conformations tested in units of Debye (D). Note that for all peptides, the carbon atom adjacent to the sulfur atom was chosen as the origin with the +z direction defined as pointing toward the C-terminal end of the peptide for the stretched and lowest energy conformations. For the ideal binding conformations, the +z-direction often pointed to a different oxygen atom as shown in **Figure S2**. Since the phosphorylated peptides possess an overall charge (–1), the choice of origin affects the dipole moment.

SAMs	z-component (D)	Magnitude (D)
Stretched		
**Control**	-125.05	125.88
**MHC1**	-193.04	195.94
**MHC2**	-237.51	237.62
**MHC3**	-273.08	273.91
Lowest Energy		
**Control**	-56.34	62.36
**MHC1**	-81.86	83.03
**MHC2**	-50.54	51.45
**MHC3**	-12.76	85.82
Ideal Binding		
**Control**	-34.90	35.56
**MHC1**	-81.86	83.03
**MHC2**	-58.67	59.50
**MHC3**	-80.33	80.40

Optimized atomic coordinates from DFT calculations for each peptide in 1) stretched conformation, 2) lowest energy conformation, and 3) ideal binding conformation.

1) Stretched conformations

[3Mpa]RQASISISV (control)

C	-0.96145898	1.30470369	5.51071373
C	-1.58014359	0.40708325	6.63229753
N	-1.53716445	0.93549350	4.19916264
O	-2.07296704	-0.67124738	6.32634734
H	2.73736287	-4.45654025	4.03238971
H	-1.20059704	2.35360500	5.71224743
H	-2.32178966	0.29913436	4.25875258
C	-1.89106774	0.12762454	9.12141244
C	-1.33394445	0.89097242	10.37496579
N	-1.45461471	0.85094751	7.91146026
O	-0.71861725	1.93763225	10.20503114
H	-7.52538693	1.59073242	8.74077711
H	-1.42992534	-0.86533402	9.11298000
H	-1.00289056	1.73536820	8.12250496
H	-1.55720460	-0.23805723	34.07909769
C	-1.04067827	0.87474008	12.87429442
C	-1.70177690	0.08721701	14.05657815
N	-1.54878829	0.33618335	11.59638144
O	-2.46538250	-0.83913627	13.80958510
H	0.90738203	1.34352430	12.09190371
H	-1.35281526	1.92068611	12.95301983
H	-2.12134820	-0.49339429	11.71090491
C	-1.94056546	-0.06129131	16.55725607
C	-1.10151630	0.46472744	17.77701450
N	-1.36580834	0.47339836	15.31460011
O	-0.25876935	1.32948986	17.58505202
H	-3.39136114	2.10919151	17.39661329
H	-1.87088029	-1.15363192	16.53804113
H	-0.74503924	1.25823655	15.47141752
C	-0.62022877	0.14503385	20.25005965
C	-1.56254173	-0.10647642	21.48031892
N	-1.36637206	-0.08849360	18.99511306
O	-2.67133040	-0.59667229	21.30262315
H	3.40197076	-0.84875704	20.60393152
H	-0.29046799	1.18720797	20.25147950
H	-2.16126312	-0.70528882	19.10992867
C	-1.73687421	-0.00186787	23.99503273
C	-0.69534436	0.18045386	25.15911106
N	-1.04861721	0.17337330	22.70769629

O	0.40347211	0.65016062	24.89194053
H	-2.04385287	2.60409442	24.74164585
H	-2.14433010	-1.01665743	24.04441537
H	-0.14494316	0.62073406	22.80682691
C	-0.27660431	-0.17321088	27.64628458
C	-1.24252284	-0.09999918	28.88778110
N	-1.08828837	-0.19220145	26.40790076
O	-2.45052578	-0.22909981	28.70608086
H	3.23817734	-2.35106928	28.07817926
H	0.35611463	0.71781090	27.62269065
H	-2.05187560	-0.45635255	26.58583334
C	-1.31848142	0.06628506	31.41830052
C	-0.19596343	0.01068796	32.52704030
N	-0.65813855	0.02770912	30.10107817
O	0.96245709	0.20886113	32.14982063
H	-0.80299583	2.58356730	32.09285264
H	-1.97179791	-0.80525767	31.52006971
H	0.34227540	0.18277851	30.19802276
C	0.56876641	1.16504633	5.56601072
C	1.11457229	-0.25941990	5.51407176
H	0.90415366	1.61913074	6.50109359
H	0.98655649	1.75953149	4.75361695
C	0.80823171	-0.97160601	4.20780953
H	0.71091020	-0.84924200	6.34449911
H	2.19945856	-0.21356600	5.64564232
N	1.49195301	-2.26344445	4.16303771
H	1.15128687	-0.36461433	3.36661727
H	-0.26469454	-1.13700260	4.10928639
C	1.76362503	-2.94224499	3.06269165
N	1.41583679	-2.48715775	1.86469617
N	2.42399932	-4.10709285	3.14352905
H	1.59278791	-2.74929685	5.04183425
H	1.75618387	-2.00892829	33.18753308
H	2.40620152	-4.75517573	2.37518463
C	-3.40958764	-0.07004586	9.13972252
H	-3.67647270	-0.70180151	9.99122208
H	-3.67994244	-0.62095142	8.23809059
C	-4.21439220	1.22088575	9.16774245
C	-5.58069953	1.03275767	8.52564588
O	-5.73038716	0.38041733	7.50730997
N	-6.60264740	1.67810009	9.13284785
H	-3.71158690	1.98198455	8.56163977
H	-4.29873950	1.62677415	10.17882508
H	-6.48539593	2.16119476	10.00482158
C	0.48119575	0.80145836	12.93516508
H	0.81413950	-0.23768875	12.90186938

H	0.85797426	1.25179691	13.85432935
C	-3.42736698	0.29867323	16.67601268
H	-3.83164706	-0.12417168	17.60349691
H	-3.95094956	-0.16727006	15.84137081
O	-3.67702275	1.68229663	16.58119393
C	0.61905109	-0.77479057	20.37729518
C	0.22353665	-2.24711302	20.38478882
H	1.05451951	-0.54158937	21.35607028
C	1.68713879	-0.46312775	19.32941785
C	3.02607492	-1.12723901	19.61571892
H	1.08729312	-2.87936279	20.59095788
H	-0.18413140	-2.54636990	19.41524730
H	-0.52813628	-2.46308988	21.14650604
H	1.82399401	0.61987123	19.27279506
H	1.33057233	-0.77037473	18.34138500
H	3.77340834	-0.82170951	18.88065465
H	2.96235075	-2.21664585	19.57987684
C	-2.91433292	0.97212629	24.13046770
H	-3.41530768	0.80176756	25.09101367
H	-3.62564977	0.75021923	23.33532616
O	-2.54299345	2.32191384	23.96703625
C	0.62332819	-1.42355744	27.79544394
C	-0.20271109	-2.70447773	27.81694148
H	1.09697057	-1.31744061	28.77818772
C	1.74981007	-1.47210378	26.76360043
C	2.81581515	-2.51091010	27.08253786
H	0.42179925	-3.56496820	28.05809276
H	-0.66281116	-2.88717230	26.84187290
H	-0.99920792	-2.66037003	28.56223368
H	2.21453140	-0.48451849	26.70014237
H	1.33251849	-1.66699667	25.77153638
H	3.63568885	-2.45579923	26.36310081
H	2.42221350	-3.52902883	27.04919020
C	-2.18442223	1.31767896	31.57836493
H	-2.66938869	1.28321855	32.56111016
H	-2.95933218	1.30517616	30.81180433
O	-1.46745195	2.52087046	31.39631954
C	0.26712235	-0.27798747	35.02674820
C	-0.64175644	-0.05450946	36.36022982
N	-0.57414758	-0.22262593	33.79727824
O	-1.87014887	0.00974472	36.11743595
H	-0.89538115	2.16322997	0.28096121
H	0.99761265	0.53309865	34.97506046
H	-2.35553723	-0.03118550	1.87223334
H	1.82481831	-2.89959277	1.04341492
H	0.88603785	-1.62903299	1.72308554

O	0.00000000	-0.00000000	37.41306422
S	0.00000000	0.00000000	0.00000000
C	-1.35398382	1.17766437	0.34834179
C	-2.00064284	0.99630658	1.75030297
C	-1.14515933	1.41743174	2.97276365
H	-2.89717466	1.62712245	1.78539479
O	-0.18894317	2.16288536	2.82630751
H	-0.77554118	-0.98899203	-0.48031441
H	-2.10381249	1.07890302	-0.43202711
C	1.01633363	-1.60833055	35.19817019
C	0.06430151	-2.79741287	35.23800444
C	2.13960544	-1.84591417	34.19706349
H	1.45908785	-1.50347562	36.19330150
H	-0.42343603	-2.94330512	34.26882253
H	-0.71389544	-2.65619704	35.99013273
H	0.60881667	-3.71570695	35.47543366
H	2.71706464	-2.73085034	34.48424613
H	2.82032686	-0.99334379	34.14959438

[3Mpa]RQA[pS]ISISV (MHC1)

C	-1.20157849	1.38700024	5.44848926
C	-1.74452756	0.42644472	6.56660378
N	-1.68893027	0.92444894	4.12807125
O	-2.20695490	-0.66156284	6.23192328
H	3.13628787	-3.94083442	4.33962919
H	-1.59297213	2.39281790	5.62787418
H	-2.39135766	0.19752222	4.20187036
C	-1.93114493	0.09105704	9.06888155
C	-1.18958851	0.78683666	10.26376564
N	-1.60006554	0.84567175	7.84150549
O	-0.56672247	1.82358524	10.03873056
H	-6.56752742	0.94070931	11.98530702
H	-1.54755842	-0.92647685	8.94920586
H	-1.15107905	1.73179179	8.06071917
H	-1.61538756	-0.04902469	33.96368866
C	-0.87250352	0.80071096	12.76041078
C	-1.38217009	-0.10640579	13.92368524
N	-1.31062083	0.21241649	11.47896374
O	-1.90333153	-1.18630478	13.67784129
H	0.97541778	1.58841961	11.98707849
H	-1.37448210	1.76536571	12.87881718
H	-1.88935677	-0.61124482	11.61261590
C	-1.74703573	-0.17436664	16.40364462
C	-0.98715713	0.38725760	17.65541754
N	-1.13826413	0.35814662	15.17941085

O	-0.14168178	1.25627483	17.52140548
H	2.59867861	-1.40887234	34.05035171
H	-1.61934732	-1.26074745	16.40466957
H	-0.84179396	1.31922806	15.27796202
C	-0.55266527	0.05979662	20.12394137
C	-1.48191321	-0.16099646	21.36644559
N	-1.27993813	-0.18907581	18.86833531
O	-2.58981601	-0.66012628	21.23290465
H	3.47193051	-0.95849303	20.48610060
H	-0.21616904	1.10033680	20.10912533
H	-2.09746795	-0.77654049	18.96151762
C	-1.65394987	0.02503932	23.87053030
C	-0.63014446	0.15638380	25.05385142
N	-0.95275642	0.15411775	22.58820476
O	0.50141584	0.55910631	24.82323999
H	-1.81090783	2.60923622	24.66871401
H	-2.11855353	-0.96516829	23.91602093
H	-0.05593887	0.61532700	22.65892684
C	-0.28064934	-0.21668378	27.54590738
C	-1.22827871	-0.01869265	28.78731323
N	-1.07717278	-0.17897149	26.30097320
O	-2.44364265	-0.02326176	28.61997985
H	2.98941963	-2.75658292	27.98631458
H	0.44143302	0.60335746	27.50611623
H	-2.05853881	-0.38100835	26.45070306
C	-1.29020310	0.22271745	31.30960622
C	-0.20644846	0.03519478	32.44197469
N	-0.62872119	0.07201863	30.00127609
O	0.97534194	0.09800006	32.09412474
H	-0.40908614	2.62542302	31.93426590
H	-2.06080783	-0.54652357	31.41270035
H	0.38131135	0.12205176	30.09083147
C	0.32782018	1.46428320	5.54592990
C	1.05799703	0.12580030	5.57223880
H	0.57167731	1.99421602	6.46909180
H	0.68944604	2.07597680	4.71949743
C	0.84383234	-0.69267988	4.31210756
H	0.73574120	-0.45977415	6.44050672
H	2.12793844	0.32100023	5.69135504
N	1.68812016	-1.88734109	4.33853231
H	1.10931102	-0.09721202	3.43477508
H	-0.19904558	-1.00020524	4.23108949
C	2.01827824	-2.60528849	3.28494056
N	1.57382381	-2.29878992	2.06973579
N	2.84819659	-3.65762131	3.41784618
H	1.90055799	-2.26781412	5.24917103

H	1.38788471	-2.25317334	33.09617624
H	2.81697226	-4.38879540	2.72695357
C	-3.43526712	0.01156060	9.34943513
H	-3.57902814	-0.73946189	10.13257918
H	-3.94820791	-0.36976936	8.46533322
C	-4.07088629	1.32394111	9.80156903
C	-5.49262397	1.05281061	10.27305730
O	-6.35752232	0.70154363	9.47227040
N	-5.67526217	1.19064425	11.58936113
H	-4.12200797	2.02977651	8.96940536
H	-3.49170764	1.78110556	10.60685454
H	-4.93065573	1.45131116	12.24857727
C	0.64220034	0.95823576	12.81038128
H	1.12976150	-0.01667456	12.73624066
H	0.94826984	1.41911062	13.75010699
C	-3.25722495	0.10946619	16.41096451
H	-3.67901003	-0.13010800	17.39439274
H	-3.74033662	-0.54083075	15.67892106
O	-3.49512508	1.46568725	16.11645230
C	0.68692708	-0.86062686	20.27411139
C	0.28655452	-2.33142730	20.30671141
H	1.12720072	-0.61192482	21.24736123
C	1.75193547	-0.57339432	19.21631421
C	3.08724158	-1.24489500	19.50297995
H	1.14783730	-2.96543216	20.52045073
H	-0.12692970	-2.64010193	19.34296295
H	-0.46558288	-2.53098495	21.07264909
H	1.89575575	0.50726169	19.14175261
H	1.38620365	-0.88962208	18.23520006
H	3.83240144	-0.95435137	18.75911646
H	3.01454829	-2.33444084	19.48022711
C	-2.77955961	1.06034446	23.99480028
H	-3.32480370	0.89579968	24.93290178
H	-3.47300051	0.89980922	23.16964920
O	-2.32706030	2.39079425	23.88482226
C	0.48843471	-1.54967910	27.71747563
C	-0.46333937	-2.73954809	27.76005592
H	0.97709558	-1.47856929	28.69626014
C	1.59502943	-1.72686506	26.67823577
C	2.54336061	-2.87431375	26.99488034
H	0.07034823	-3.65491699	28.01707696
H	-0.93911067	-2.89148053	26.78739985
H	-1.25148991	-2.60157770	28.50274709
H	2.16349610	-0.79612152	26.60365873
H	1.15060334	-1.88157111	25.69106870
H	3.35751710	-2.91321397	26.26785232

H	2.03991475	-3.84308493	26.97046642
C	-1.97441501	1.58639904	31.43710475
H	-2.47661262	1.63589951	32.41102330
H	-2.72951049	1.67040147	30.65545168
O	-1.09203633	2.67421413	31.25440262
C	0.18589604	-0.32180819	34.94390620
C	-0.66426521	0.03232008	36.29099786
N	-0.63310530	-0.15174728	33.70800311
O	-1.87490914	0.26576068	36.07163637
H	-1.10631727	2.07746778	0.17554986
H	1.02824366	0.37163196	34.88594907
H	-2.37983399	-0.18966061	1.82495668
H	2.02977141	-2.70216324	1.26889735
H	0.98125144	-1.49234460	1.88684948
O	0.00000000	0.00000000	37.33214070
S	0.00000000	0.00000000	0.00000000
C	-1.46994348	1.05586615	0.27454557
C	-2.11998583	0.86221624	1.67393979
C	-1.32844923	1.40418871	2.89528868
H	-3.07140997	1.40780531	1.67502230
O	-0.45124809	2.23937129	2.72712412
H	-0.66593071	-1.07306963	-0.46359347
H	-2.19078389	0.85231004	-0.51278203
C	0.73139071	-1.74869569	35.11292689
C	-0.38801618	-2.78223096	35.15970249
C	1.79876650	-2.15049781	34.10300505
H	1.19277088	-1.71131540	36.10446919
H	-0.89992666	-2.85011436	34.19438834
H	-1.13053400	-2.52729261	35.91818039
H	0.01425949	-3.77274493	35.39059807
H	2.23922342	-3.11280810	34.38478604
P	-4.52873666	1.79234030	14.86675889
O	-3.72375221	1.98360509	13.60303981
O	-5.01849305	3.25680526	15.36724057
O	-5.65354438	0.83065114	14.89241993
H	-4.49827080	3.90854922	14.88525229

[3Mpa]RQASI[pS]ISV (MHC2)

C	-0.89503710	-1.30274638	5.54628569
C	0.03938940	-1.80542440	6.69667729
N	-0.28573209	-1.66697594	4.24805869
O	0.95555483	-2.57167198	6.43176456
H	2.43923574	4.73525813	3.85222137
H	-1.80389084	-1.90012710	5.67819740
H	0.44290479	-2.36527648	4.36386679

C	0.27893538	-1.96994008	9.19346733
C	-0.07653906	-1.01509449	10.38786888
N	-0.26034906	-1.39188286	7.95567396
O	-0.48586132	0.11334222	10.13591248
H	-3.78521412	-5.52377003	7.74147739
H	1.37197919	-1.96739572	9.12547265
H	-0.92648144	-0.64929059	8.11185417
H	0.56581286	1.17041179	33.92570245
C	0.04651423	-0.63569166	12.86403454
C	-0.15887787	-1.56488945	14.10814191
N	0.15681072	-1.46362532	11.64636656
O	-0.43820240	-2.74656783	13.92407664
H	1.32472830	0.90131123	12.07601459
H	-0.86611224	-0.03845699	12.77652272
H	0.27082817	-2.45170485	11.83418246
C	-0.41146245	-1.62305630	16.60615241
C	0.24340677	-0.80726674	17.78419144
N	-0.07128223	-0.98500642	15.32698481
O	0.99988601	0.11051370	17.50394534
H	-2.45593182	-0.15229554	17.67543032
H	0.02894222	-2.62586853	16.62188936
H	0.32354485	-0.05949300	15.44284070
C	0.68870234	-0.95833758	20.28657584
C	-0.22867064	-0.61158387	21.52959157
N	-0.08913965	-1.20191853	19.04189812
O	-1.42303629	-0.38431656	21.36978730
H	4.33588295	-2.74398045	20.71750305
H	1.30022301	-0.07278589	20.09875290
H	-0.73194309	-1.97593054	19.11186481
C	0.00590062	0.11189578	23.93185732
C	-0.14260240	-0.79754473	25.21742962
N	0.43856005	-0.58663212	22.71229493
O	-0.20301010	-2.00819781	25.12961952
H	-2.89466604	-1.46412768	34.44368192
H	-0.98525837	0.53405487	23.73799981
H	1.47729050	-0.60607175	22.63901842
C	-0.52031415	-0.72374309	27.72804531
C	0.01702482	0.23887977	28.84693484
N	-0.30459803	-0.11562457	26.40627440
O	0.38383607	1.37181485	28.55599131
H	-3.87330339	-3.01619473	28.58595873
H	0.03459057	-1.66606610	27.77230432
H	0.01965276	0.84074582	26.47549213
C	0.34107571	0.53797184	31.32843676
C	-0.21620079	-0.21839315	32.59504644
N	-0.00630512	-0.22908706	30.12512142

O	-0.78709614	-1.29274287	32.41466531
H	2.45421775	-0.68190865	32.50989443
H	-0.15254680	1.51468174	31.27321565
H	-0.36781372	-1.14967298	30.34434804
C	-1.29444288	0.16628554	5.66073638
C	-0.16960445	1.19292548	5.57838243
H	-1.81189530	0.29841767	6.61335093
H	-2.03552599	0.37306863	4.88841505
C	0.37085747	1.35228953	4.16806534
H	0.64218400	0.93261800	6.26635485
H	-0.57019490	2.15789860	5.90476276
N	1.20124336	2.55318522	4.07857643
H	-0.45989789	1.44907617	3.46461218
H	0.96308773	0.48224961	3.88036789
C	1.56438393	3.13026736	2.94997247
N	1.28870525	2.57314167	1.77663487
N	2.19649791	4.31721189	2.96992856
H	1.55259449	2.93810937	4.94237066
H	-2.90669205	-0.04245637	33.41125221
H	2.76459497	4.58360444	2.18335161
C	-0.17172191	-3.42045595	9.36710773
H	0.34440487	-3.84773684	10.22989152
H	0.18715432	-3.97103942	8.49603421
C	-1.68605941	-3.60869434	9.51877415
C	-2.34719092	-4.19176254	8.28317126
O	-2.07359755	-3.82942370	7.14854282
N	-3.28716588	-5.13205053	8.52315819
H	-2.17845768	-2.64648698	9.69788232
H	-1.90859916	-4.22221122	10.39350519
H	-3.50881679	-5.44211272	9.45179576
C	1.24109990	0.30115232	12.98232681
H	2.16535484	-0.26121115	13.12561653
H	1.12511311	0.99040114	13.81995078
C	-1.92642966	-1.77318493	16.74883583
H	-2.15428538	-2.39748998	17.62151793
H	-2.30088335	-2.30328370	15.87186009
O	-2.60669846	-0.53990856	16.80368834
C	1.62982630	-2.14790515	20.56051732
C	0.86031086	-3.42472893	20.87699304
H	2.20865720	-1.86328685	21.44145620
C	2.64984901	-2.33194770	19.43610597
C	3.84041673	-3.18520086	19.84976341
H	1.54400065	-4.22461694	21.16528661
H	0.29160312	-3.78836317	20.01203652
H	0.17240590	-3.27526699	21.71191051
H	3.01384964	-1.34651296	19.13207296

H	2.16679040	-2.77373091	18.55405783
H	4.57284399	-3.25479427	19.04137588
H	3.54444876	-4.20385320	20.11268998
C	1.00744121	1.24219452	24.19920690
H	0.61055231	1.94089540	24.94324805
H	1.16532455	1.81528122	23.28020434
O	2.21649375	0.70858466	24.69699965
C	-2.01375801	-1.01671961	28.01579825
C	-2.84445194	0.26095488	27.98122747
H	-2.05045705	-1.41213126	29.03845544
C	-2.57937850	-2.10211304	27.10107496
C	-3.92741955	-2.63820715	27.56067856
H	-3.87787444	0.06431409	28.27067211
H	-2.84674425	0.68746474	26.97489069
H	-2.45044728	1.01703890	28.66362600
H	-1.86162018	-2.92377241	27.04275822
H	-2.66131689	-1.71617039	26.08152451
H	-4.25409834	-3.46124781	26.92049757
H	-4.70779383	-1.87402269	27.53098296
C	1.84377203	0.80415006	31.42645362
H	2.02573071	1.53247102	32.22664543
H	2.17133370	1.25067589	30.48773076
O	2.61683965	-0.36339106	31.61436241
C	-0.54956203	-0.10746668	35.12120076
C	0.31801484	0.46857818	36.36381594
N	-0.04448134	0.36550235	33.80441566
O	1.16877660	1.32786034	36.03707994
H	-1.65797456	-1.64026406	0.31718493
H	-0.47921158	-1.19688433	35.13438438
H	0.91645238	-2.15473260	1.92802051
H	1.37138788	3.12326569	0.93870013
H	0.82509708	1.67096542	1.68042092
O	0.00000000	0.00000000	37.46469444
S	0.00000000	0.00000000	0.00000000
C	-0.57323097	-1.68803041	0.39944024
C	-0.17149511	-2.17740124	1.81606200
C	-0.83772729	-1.45453227	3.01245589
H	-0.45722823	-3.23333901	1.89698810
O	-1.80079596	-0.72259539	2.82451968
H	1.23163198	-0.34445080	-0.41715904
H	-0.18862746	-2.36518496	-0.35920389
C	-2.01363628	0.29620616	35.36831423
C	-2.19657294	1.80979260	35.34965445
C	-3.01807381	-0.37937692	34.44464213
H	-2.19345459	-0.04960689	36.39047317
H	-2.01462650	2.21425098	34.34877841

H	-1.50935288	2.30066031	36.04115986
H	-3.21990601	2.07560490	35.63154373
H	-4.03995468	-0.14641557	34.76284999
P	3.51732897	0.64589258	23.67737441
O	4.06911752	2.00779072	23.48158667
O	3.12083119	-0.21902360	22.51327454
O	4.49256304	-0.24279467	24.61978164
H	5.00587446	0.37493915	25.15323436

[3Mpa]RQASISI[pS]V (MHC3)

C	-0.14247759	-1.52224779	5.54930927
C	0.94325544	-1.71252259	6.66049057
N	0.49971768	-1.68348305	4.23589921
O	2.10124726	-1.94158334	6.34338160
H	1.10529163	5.27567141	3.82213163
H	-0.86790846	-2.33286507	5.68295285
H	1.45976802	-2.00421790	4.30604665
C	1.35929813	-1.87225917	9.13168057
C	0.62740632	-1.28318257	10.38416475
N	0.51183457	-1.64874298	7.95020021
O	-0.28769436	-0.48767105	10.20889738
H	0.99613072	-7.67924062	9.12969527
H	2.26185173	-1.25875896	9.02317917
H	-0.38916807	-1.24439694	8.17160997
H	1.73749604	-0.43741669	34.01396859
C	0.69746655	-0.91110232	12.85143460
C	0.84107016	-1.84170691	14.09936138
N	1.10575046	-1.60491413	11.61497376
O	0.87266932	-3.05572192	13.94792492
H	1.27115542	0.99584313	12.04923700
H	-0.37681369	-0.72198266	12.76157014
H	1.72896229	-2.39093510	11.72392506
C	0.63964578	-1.92279272	16.59436814
C	1.22772647	-1.08207358	17.78280589
N	0.84784555	-1.23418975	15.31651296
O	1.88364250	-0.08211174	17.55355781
H	-1.52856503	-0.70746185	17.76584601
H	1.18686285	-2.87052511	16.55846673
H	0.94438596	-0.23247139	15.38676628
C	1.66178887	-1.19325503	20.27711954
C	0.69419964	-0.76012325	21.44928279
N	0.94722871	-1.55630723	19.03565842
O	-0.35952407	-0.20563909	21.17420067
H	5.40651703	-2.90212371	20.92514527
H	2.19617064	-0.27060019	20.03287280

H	0.43256416	-2.42216463	19.08059724
C	0.53598337	-0.34463018	23.92195701
C	0.99923419	-1.15539590	25.18898268
N	1.14876771	-0.92370064	22.72103835
O	1.66111717	-2.17014495	25.02193629
H	2.45211969	1.18403125	25.13347035
H	-0.55132574	-0.44773234	23.84278740
H	1.91246307	-1.55044727	22.93548720
C	0.54094441	-1.37436905	27.68663965
C	0.92160285	-0.44373126	28.90692232
N	0.61644943	-0.65165218	26.39093007
O	1.33676865	0.69449503	28.70599939
H	-2.10710628	-4.48145443	28.08747433
H	1.28278562	-2.17509672	27.65039637
H	0.10751463	0.21909110	26.38201885
C	1.33017499	-0.63037372	31.39811363
C	0.29486112	-0.24409526	32.53329154
N	0.73303733	-1.03002486	30.11326498
O	-0.87349008	-0.03450898	32.25579651
H	-1.79421004	2.10665721	33.94875509
H	1.93982491	0.26083510	31.21943208
H	0.55576047	-2.05240242	30.07004453
C	-0.91515150	-0.20841299	5.70247546
C	-0.10963367	1.08124401	5.56923252
H	-1.39306084	-0.21387440	6.68493841
H	-1.72499711	-0.21444596	4.97154647
C	0.23349418	1.42958280	4.12902828
H	0.80516792	1.03060865	6.17021436
H	-0.71362283	1.89618252	5.97900607
N	0.67246954	2.81655922	4.03357748
H	-0.65430572	1.30973685	3.50256790
H	1.00824525	0.76247778	3.74533130
C	0.72413055	3.51253307	2.90026307
N	0.58356826	2.92439187	1.70240506
N	0.96125275	4.84881737	2.91955601
H	1.11792200	3.22007656	4.84650229
H	-0.32289024	2.44826984	33.05444143
H	1.58038644	5.20270587	2.19827552
C	1.81381598	-3.32849361	9.21230942
H	2.55280795	-3.43610149	10.01197432
H	2.33891800	-3.54999179	8.28147702
C	0.69062534	-4.34328167	9.37674261
C	1.07285661	-5.68135551	8.76340324
O	1.60310248	-5.75777034	7.66890350
N	0.74286865	-6.77414724	9.48984816
H	-0.18687337	-4.00807986	8.81418433

H	0.38223792	-4.44821697	10.41974631
H	0.39562494	-6.70187466	10.42907494
C	1.41699794	0.42698955	12.96784133
H	2.48671361	0.28879267	13.13451982
H	1.01217795	1.02998797	13.78228510
C	-0.84710221	-2.24053768	16.78350954
H	-0.98591898	-2.90548381	17.64514211
H	-1.18969303	-2.78902953	15.90493848
O	-1.64687336	-1.08585014	16.88499815
C	2.69531122	-2.27390257	20.64522904
C	2.04396974	-3.61927888	20.95547248
H	3.19783918	-1.91629436	21.55066044
C	3.78530891	-2.38835220	19.57725621
C	4.96346371	-3.25751901	19.99130020
H	2.76375059	-4.31111473	21.39302697
H	1.65778287	-4.09258619	20.04631589
H	1.22429295	-3.52219274	21.67017716
H	4.14519880	-1.38355424	19.33593480
H	3.34894000	-2.78518233	18.65340739
H	5.74375798	-3.24066574	19.22753915
H	4.67334417	-4.29955307	20.13864271
C	0.86218470	1.14606135	24.02283525
H	0.25358077	1.60724927	24.81022630
H	0.58085131	1.62128531	23.08188187
O	2.23558388	1.40324302	24.21799710
C	-0.85110515	-2.01440842	27.86426698
C	-1.94954483	-0.96388073	27.97266072
H	-0.79665127	-2.56183903	28.80724563
C	-1.12938750	-3.06333203	26.78799039
C	-2.29400032	-3.97941344	27.13538195
H	-2.90987473	-1.42985696	28.19888774
H	-2.07438957	-0.40557076	27.03683040
H	-1.74042406	-0.25724128	28.77870533
H	-0.23042051	-3.67195182	26.66003717
H	-1.31893532	-2.57577131	25.82267805
H	-2.42855010	-4.74858200	26.37034250
H	-3.23685105	-3.43325971	27.22051419
C	2.23068673	-1.78276751	31.85363768
H	2.81987330	-1.49137211	32.72821981
H	2.93981178	-2.03446284	31.05863589
O	1.44033931	-2.89990739	32.21027256
C	0.08902264	0.35944523	34.99190363
C	0.74560202	-0.17739457	36.40503998
N	0.82034876	-0.07282427	33.78981182
O	1.89430056	-0.63899209	36.28582181
H	-0.95140338	-2.11946773	0.37147353

H	-0.92612894	-0.03658420	34.93697514
H	1.69107323	-1.65508952	1.87735797
H	0.21850476	3.48747872	0.95137327
H	0.39847737	1.92858046	1.62297663
O	0.00000000	-0.00000000	37.37649809
S	0.00000000	0.00000000	0.00000000
C	0.08206098	-1.77689475	0.40666623
C	0.69008878	-2.08904729	1.79688752
C	-0.15236080	-1.69046263	3.03038037
H	0.83014734	-3.17500780	1.86352505
O	-1.33493167	-1.40464498	2.91499713
H	1.26141254	0.11868657	-0.45180460
H	0.64694257	-2.28111937	-0.37361750
C	0.01932271	1.89261042	35.13046107
C	1.40224082	2.52652523	35.22378483
C	-0.80178575	2.55335963	34.03017913
H	-0.49200207	2.04652305	36.08530096
H	1.95770204	2.38061493	34.29240046
H	1.98852639	2.09053080	36.03462189
H	1.31782501	3.60372010	35.39627429
H	-0.91447301	3.62315824	34.23856763
P	1.42642695	-4.19428161	31.18694576
O	2.77046388	-4.82307438	31.15455084
O	0.72444257	-3.76386150	29.93031667
O	0.37775475	-5.11876641	32.01472703
H	0.89329111	-5.60876410	32.66542908

2) lowest energy conformations

[3Mpa]RQASISISV (control)

C	-0.90620381	3.54147168	3.20080506
C	-1.87256372	2.54320197	3.84224489
N	0.45216507	3.25275309	3.60943744
O	-1.60083338	1.88560293	4.83824883
H	-5.29111321	4.79474216	1.66710996
H	-0.98402761	3.41287243	2.11534253
H	0.79555006	3.77716927	4.40389989
C	-3.75347986	1.15751961	3.35391382
C	-4.43814274	1.03818229	4.71158120
N	-3.05207472	2.42667944	3.19747974
O	-4.73633925	-0.05463128	5.16666677
H	-4.44327813	-2.03940925	4.01522660
H	-3.00473168	0.35316113	3.32072332
H	-3.16671818	2.90723390	2.28518113
H	-1.54222405	0.26954505	13.48136208
C	-5.26553169	2.25589310	6.65901419
C	-4.22524037	2.60032894	7.73703931
N	-4.68557459	2.20521063	5.32928842
O	-4.57459420	2.84651937	8.87274496
H	-6.78005049	3.32191391	7.73285682
H	-5.63663676	1.24691679	6.88444149
H	-4.38129302	3.06475905	4.89546589
C	-1.85212622	2.80019751	8.24731844
C	-0.98794988	1.55514824	8.38140881
N	-2.94441049	2.58486789	7.32353908
O	0.03114003	1.57478486	9.07346710
H	-0.38856644	2.99684175	6.21443567
H	-2.28083696	3.01858247	9.23395968
H	-2.69860892	2.40686667	6.35817994
C	-0.77639150	-0.81160883	7.98792725
C	-1.59253233	-1.42573386	9.13148892
N	-1.37926913	0.47615049	7.69211236
O	-2.64292034	-0.92628895	9.49523139
H	2.05934474	-0.37467365	5.09728475
H	0.23861670	-0.61967574	8.35530598
H	-2.32415638	0.45637818	7.33293131
C	-1.56883148	-3.27003856	10.80622025
C	-0.47113412	-4.03692884	11.55861743
N	-1.00822741	-2.49450107	9.70265647
O	-0.70066519	-5.17721244	11.90791487
H	-2.07189131	-0.58249009	11.33401368
H	-2.23192618	-4.04561690	10.39594451

H	-0.15890755	-2.86274654	9.30393200
C	1.06720984	-2.05362722	11.80166920
C	1.13976659	-1.65989398	13.28994689
N	0.73566442	-3.46906619	11.78551381
O	1.61067515	-2.41508490	14.11453139
H	4.13686144	1.15015782	10.39565463
H	0.29240740	-1.48425504	11.28462939
H	1.30001398	-4.00809069	12.43643230
C	0.57052119	0.21154040	14.83541441
C	-0.36826038	1.40974079	14.70335315
N	0.64548290	-0.43511125	13.53189943
O	-0.16191981	2.45601086	15.28192757
H	-0.27098113	0.92005745	17.02285495
H	1.56014590	0.57650728	15.13871695
H	0.29507732	0.10164710	12.75219633
C	-1.27400135	4.98574695	3.56267390
C	-0.57892462	6.01159482	2.66097013
H	-2.35908256	5.10143875	3.49200601
H	-0.98993062	5.17237528	4.60097142
C	-1.06220909	6.00563175	1.20113876
H	0.49937568	5.82750977	2.66341602
H	-0.75110511	7.00671554	3.08210882
N	-2.50341585	6.21426984	1.10597425
H	-0.56970494	6.82086864	0.66234533
H	-0.80457449	5.06870146	0.70175871
C	-3.36672987	5.14925773	1.25486940
N	-3.15372282	3.93768479	0.90160787
N	-4.56317240	5.46471974	1.85629145
H	-2.80558156	7.05040942	1.59008429
H	-4.22664617	3.96936141	12.79384146
H	-4.85227754	6.42629831	1.79634242
C	-4.74629173	0.97354689	2.19925906
H	-4.22824919	1.19839878	1.26215190
H	-5.58412938	1.66800378	2.30622500
C	-5.24456676	-0.47448828	2.15471065
C	-4.02333241	-1.38725333	2.12352377
O	-3.20510921	-1.34073451	1.22709698
N	-3.91672932	-2.22307772	3.17863377
H	-5.87992630	-0.69469102	3.01375016
H	-5.81758355	-0.62800101	1.23791908
H	-3.06345666	-2.74829963	3.25350033
C	-6.41552638	3.25896791	6.70831973
H	-6.07323730	4.24588154	6.39471374
H	-7.22593274	2.94002618	6.05465980
C	-0.97742203	3.99190719	7.81917950
H	-0.09037645	4.01109672	8.47038944

H	-1.53710772	4.92456522	7.94655725
O	-0.61327685	3.90857754	6.45802966
C	-0.71852324	-1.70437503	6.73781330
C	-2.12523931	-2.07498264	6.25674114
H	-0.19525091	-2.62705565	7.02696706
C	0.07094213	-1.01336370	5.61397866
C	1.54044991	-0.78517646	5.96284487
H	-2.05384185	-2.78483438	5.43242660
H	-2.66427211	-1.19465257	5.90596608
H	-2.69838730	-2.53495611	7.06166858
H	-0.40246884	-0.05436421	5.38517985
H	0.00774001	-1.63968209	4.71816699
H	2.02063418	-1.72288232	6.24779845
H	1.64310051	-0.07419515	6.78271194
C	-2.41836900	-2.42808434	11.79392155
H	-2.48604119	-2.98974107	12.73285171
H	-3.42798528	-2.30763142	11.38042795
O	-1.85896629	-1.17006680	12.08196938
C	2.44106909	-1.80717696	11.13070982
C	2.53971473	-2.55904655	9.79990826
H	3.21901637	-2.19360531	11.80295302
C	2.63776269	-0.29450522	10.93982050
C	4.02552332	0.06597309	10.41171112
H	3.55845604	-2.51767072	9.41742580
H	2.26826477	-3.60572208	9.93741811
H	1.87393614	-2.11898698	9.05490180
H	2.49271687	0.20285306	11.90386732
H	1.87545348	0.08746053	10.25100061
H	4.80090060	-0.35372417	11.05387080
H	4.17007291	-0.30726184	9.39790009
C	0.01543725	-0.70683573	15.94487905
H	-0.99452427	-1.04954174	15.66231160
H	0.66445084	-1.57811332	16.07576376
O	-0.00000000	-0.00000000	17.15770751
C	-2.13904982	2.25990783	13.27569416
C	-1.44228173	2.49640318	11.93586106
N	-1.47188895	1.16234015	13.95448471
O	-1.59752750	3.48462453	11.26433812
H	0.07333325	2.27808049	0.81404136
H	-2.04360481	3.16320793	13.88985951
H	-0.59560218	0.85866021	2.77198944
H	-2.27461417	3.81131458	0.40701407
H	-5.42772009	2.77840209	12.26497855
O	-0.67354155	1.48059784	11.56395883
S	0.00000000	0.00000000	0.00000000
C	0.60049933	1.35007400	1.05232298

C	0.45936920	1.00585308	2.53548567
C	1.07081271	2.03837671	3.47205127
H	0.98061212	0.06498969	2.72576970
O	2.10275702	1.82155819	4.07011671
H	-1.19900519	-0.14092741	0.55893315
H	1.65308971	1.47874358	0.79431211
C	-3.62559951	1.90212579	13.05528804
C	-4.28324993	1.64295839	14.41426715
C	-4.36258785	3.00753908	12.29805973
H	-3.66449587	0.97574675	12.46489166
H	-3.74155206	0.86633999	14.95450123
H	-4.28472985	2.55149523	15.01792765
H	-5.31290156	1.31577341	14.27136771
H	-4.00003254	3.08689911	11.27462787
H	-3.97251632	3.37839951	0.54316084

[3Mpa]RQA[pS]ISISV (MHC1)

C	-3.66452215	-0.73846309	4.51697690
C	-3.47001660	-1.84451851	5.58057357
N	-3.21362653	-1.18678505	3.19718111
O	-4.43840921	-2.23710133	6.19867604
H	-4.94211624	3.43254624	8.72071685
H	-4.75710701	-0.63793688	4.43704339
H	-3.81800360	-1.88718430	2.79447851
C	-0.97475740	-1.84281397	5.36449362
C	-0.32778811	-1.16602459	6.57144594
N	-2.24915127	-2.38308847	5.78643541
O	-0.49028004	-1.59310036	7.70812405
H	2.79657679	-4.12112800	4.99984199
H	-1.13062085	-1.10767748	4.58096969
H	-2.20196251	-2.97769107	6.60574762
H	1.14558244	0.20137731	14.74058494
C	1.00706267	0.74122172	7.30483408
C	1.78105292	-0.13007970	8.28897680
N	0.41641176	-0.10204423	6.26667739
O	2.58075798	-0.96744274	7.87697992
H	2.45664945	2.32433632	7.40823712
H	0.20071196	1.29234236	7.79233773
H	0.45376543	0.24303537	5.32041467
C	0.69040829	0.97029598	10.28459552
C	0.41073117	0.42430101	11.68670425
N	1.62673710	0.07800881	9.60996737
O	0.72905408	1.05189934	12.69274352
H	-1.15410653	0.15738396	14.49047821
H	-0.25698467	0.99211168	9.73387956

H	2.33102865	-0.37835865	10.18628534
C	-0.42955162	-1.73935912	10.76268649
C	0.87025448	-2.55221976	10.71398228
N	-0.24597367	-0.74288597	11.80321448
O	1.53122881	-2.71849497	11.72700821
H	-3.15014197	-3.32922023	13.34308794
H	-0.61293292	-1.22983440	9.81541117
H	-0.25081916	-1.11009438	12.74690393
C	2.54170557	-3.61515522	9.35274427
C	2.78289364	-4.59278991	10.50672968
N	1.20087511	-3.05978550	9.52051550
O	2.05763713	-5.54902340	10.67345100
H	2.47913510	-2.59078094	7.17991548
H	3.25798590	-2.78531560	9.33599918
H	0.73235263	-2.74393587	8.67927548
C	4.84313055	-3.32388625	11.23104052
C	4.18746245	-2.03011926	11.74893241
N	3.88915097	-4.41706405	11.28218047
O	3.99910983	-1.08229412	11.00141134
H	7.85909176	-2.96605137	14.04846488
H	5.12639516	-3.14281647	10.18687636
H	3.88159822	-4.98618611	12.11670371
C	3.04512883	-0.94340568	13.55146604
C	2.30685613	-1.48557787	14.76857375
N	3.89914829	-2.00850483	13.05645126
O	2.61858943	-2.53585705	15.29923003
H	2.37795450	1.56396648	13.57939566
H	2.32355152	-0.69380188	12.76754808
H	3.86066208	-2.85893528	13.60728669
C	-3.11282431	0.60554124	5.00531362
C	-3.71053022	1.78251912	4.23184055
H	-3.38796349	0.69595128	6.06093525
H	-2.02592152	0.64822546	4.94666717
C	-3.07909335	3.11124137	4.67533416
H	-4.78959202	1.82919152	4.40729829
H	-3.53467458	1.65978353	3.16023259
N	-3.07453374	3.24245246	6.11498503
H	-2.03965792	3.16875420	4.34290179
H	-3.64826451	3.93789137	4.23570254
C	-4.21152504	3.31413630	6.82761244
N	-5.35968332	3.33263102	6.19104869
N	-4.11145258	3.36025704	8.16424641
H	-2.16387768	3.29914794	6.58595415
H	-1.98578279	0.37322600	16.03757210
H	-3.20747026	3.31742571	8.62975721
C	-0.07406816	-2.95714843	4.81487055

H	0.21307066	-3.64149312	5.61695729
H	-0.64216045	-3.51758517	4.06664423
C	1.17518357	-2.34705549	4.16721951
C	1.84765741	-3.41663996	3.32274994
O	1.60899583	-3.54723133	2.13772652
N	2.68719435	-4.21474173	4.00447300
H	0.88483568	-1.52562130	3.50755755
H	1.85133476	-1.96550241	4.93417231
H	3.11027403	-4.98433060	3.51934840
C	1.98828332	1.70653543	6.64504338
H	2.75836166	1.15041788	6.11033438
H	1.45317705	2.35539529	5.95328357
C	1.23458614	2.41470685	10.37684681
H	0.76709310	2.90736790	11.23858881
H	2.32109705	2.39007174	10.52404421
O	0.96652512	3.11553826	9.19490881
C	-1.63492521	-2.64414599	11.10503585
C	-2.07849438	-3.39550827	9.84719233
H	-2.44847828	-1.97855089	11.42496628
C	-1.30236629	-3.61766464	12.24947186
C	-2.55847405	-4.15074720	12.93531338
H	-1.27907001	-4.04497858	9.48849352
H	-2.95339071	-4.00752295	10.06336799
H	-2.33610939	-2.69195610	9.05557019
H	-0.71105523	-4.45219707	11.86062155
H	-0.68618609	-3.10632961	12.99601410
H	-2.27635043	-4.81379585	13.75332708
H	-3.17903324	-4.71156012	12.23594025
C	2.59195182	-4.37201076	8.02097757
H	3.60423350	-4.78979094	7.89404934
H	1.86963723	-5.19809759	8.03887304
O	2.26789263	-3.52074372	6.94257270
C	6.09758888	-3.70555937	12.03960654
C	6.75316632	-4.94001231	11.41134841
H	5.78851380	-3.95287306	13.06484333
C	7.06456291	-2.51044350	12.08585621
C	8.22941244	-2.73319662	13.04917273
H	6.02611121	-5.74672901	11.31845391
H	7.13839320	-4.70404465	10.41832160
H	7.57645182	-5.29078076	12.03200416
H	6.51201989	-1.62266982	12.40870090
H	7.44976095	-2.31397654	11.07981800
H	8.86809592	-3.55058233	12.71445550
H	8.83347223	-1.82758131	13.10578572
C	3.87658064	0.32248232	13.86205406
H	4.57463734	0.12127988	14.68282990

H	4.45323502	0.57758928	12.95884651
O	3.04989927	1.37996194	14.25953719
C	0.47447576	-1.06456139	16.30704465
C	0.36795253	0.10396519	17.27199524
N	1.27968220	-0.70982140	15.15811116
O	-0.00000000	0.00000000	18.41088338
H	0.05504265	-2.00101085	1.34759665
H	0.96969454	-1.89999038	16.81835934
H	-2.68446242	-0.97788000	0.42557250
H	-6.15262600	3.35157360	6.83352932
H	-0.35494974	5.65627150	9.79115748
O	0.68635751	1.27404637	16.72074433
S	0.00000000	0.00000000	0.00000000
C	-0.61880207	-1.59258444	0.59065084
C	-2.04914070	-1.52037188	1.13250659
C	-2.13797080	-0.80924456	2.47894291
H	-2.44745157	-2.53450483	1.23034474
O	-1.30700503	0.00776758	2.84902050
H	-0.02482745	0.61170764	1.17606188
H	-0.59924866	-2.25316300	-0.27712263
C	-0.93464385	-1.51917489	15.84922344
C	-1.68969708	-2.18648721	16.99886718
C	-1.73203576	-0.35083836	15.26280768
H	-0.77208952	-2.26274926	15.05778252
H	-1.13131208	-3.04702171	17.36741219
H	-2.66443527	-2.52753915	16.64965783
H	-1.83801292	-1.48895676	17.82271456
H	-2.65532076	-0.72066067	14.81672274
P	-0.49963303	3.69672522	8.65395054
O	-0.34731144	5.29540066	8.90774503
O	-1.60190904	3.11297381	9.48415667
O	-0.52653248	3.50407995	7.17898987
H	-5.52207318	2.56853034	5.62087131

[3Mpa]RQASI[pS]ISV (MHC2)

C	-0.90571547	2.40482343	4.55680254
C	-0.23041326	3.78396161	4.53923300
N	-1.82770861	2.43088420	3.43425615
O	-0.86548406	4.78831803	4.26731082
H	3.67252111	1.49468791	7.87976385
H	-0.15803649	1.62141061	4.41003975
H	-2.18364659	3.36518757	3.25000032
C	1.88502092	4.97188590	4.71860941
C	3.33505554	4.59439769	4.99633362
N	1.07939662	3.76867052	4.82693149

O	3.76201386	3.46457031	4.80918228
H	-0.95563454	3.67495908	1.13329697
H	1.51374176	5.70812003	5.44024117
H	1.57926865	2.88015828	4.87616390
H	1.72287787	-0.48628823	12.23588476
C	3.83804291	6.92460376	5.81330458
C	3.03001461	6.90923948	7.13060554
N	4.18212332	5.59104919	5.34274066
O	1.90280522	7.36485501	7.10622097
H	5.52750594	7.89943138	4.89967988
H	3.16070192	7.38808177	5.08436880
H	5.13548203	5.29008325	5.48153136
C	4.72623137	5.67697634	8.57132694
C	4.81961029	5.55815177	10.08925749
N	3.57238308	6.50048704	8.29564721
O	3.84742961	5.70969719	10.81102131
H	1.96307844	-3.03412146	8.58009639
H	5.64226251	6.11337749	8.16158774
H	2.89198414	6.47167327	9.05130143
C	6.17705555	4.58445994	11.84305615
C	6.41774248	3.10487113	11.53058663
N	6.04512081	5.22148003	10.53621005
O	6.97477194	2.77062478	10.49830940
H	5.80887775	6.76953141	14.41315224
H	5.23297348	4.71368913	12.37918461
H	6.73214472	4.89570400	9.86634341
C	6.03548310	0.82463550	12.15720562
C	5.97331925	-0.12367077	13.35709245
N	5.99607332	2.24296674	12.47586386
O	6.72396341	-1.08103990	13.34900677
H	2.77230653	3.51008150	8.45532166
H	7.01048621	0.63376671	11.68945778
H	5.29779783	2.56035082	13.12793283
C	3.91878211	0.82480078	14.55159916
C	2.75364371	-0.17514028	14.55277536
N	5.14398936	0.06200920	14.41182945
O	2.91942695	-1.34883481	14.82521801
H	3.85512733	3.78663835	14.22093560
H	3.80221992	1.53606508	13.73526727
H	5.13952561	-0.76544032	15.00206849
C	0.31174870	-0.34331023	14.36012195
C	0.17757908	-1.38125006	13.23655146
N	1.55754703	0.39568980	14.31639879
O	-0.67166817	-2.24682174	13.29022803
H	-0.07891734	-0.38901736	17.56782306
H	-0.49145773	0.38992460	14.21489671

H	1.51395350	1.32349691	13.92026009
C	-1.67495963	2.18565173	5.87127782
C	-0.72993159	1.97411552	7.05524360
H	-2.30382825	3.06041969	6.06047022
H	-2.33103130	1.31876291	5.75148188
C	0.00603183	0.61768090	6.97420568
H	0.00618921	2.78305551	7.07930986
H	-1.29999863	2.01892819	7.98613718
N	1.38954396	0.78999432	7.34886528
H	-0.02007051	0.20895337	5.96144312
H	-0.44671115	-0.10600513	7.65568535
C	2.33678403	1.07476057	6.43999748
N	2.03569169	1.10291725	5.16646825
N	3.59735056	1.27456837	6.89712633
H	1.59280627	0.93842959	8.33209930
H	3.38031037	-3.83754375	9.26567621
H	4.17838958	1.82289432	6.28061417
C	1.79826032	5.57784594	3.30061194
H	0.79992633	6.00060172	3.16589416
H	2.51950164	6.39679774	3.23500181
C	2.10009008	4.55973798	2.19160012
C	0.93225072	3.64259691	1.85686646
O	0.99755690	2.43141479	2.00713467
N	-0.14265237	4.24375964	1.32080016
H	2.94678897	3.92996606	2.47351714
H	2.36198721	5.10601652	1.28115804
H	-0.25204762	5.23978927	1.34794228
C	5.09919721	7.78065369	5.89488454
H	4.84352746	8.76413260	6.28701395
H	5.85143741	7.33037530	6.54114350
C	4.53504964	4.21558004	8.02359427
H	4.90581057	3.50271011	8.77508045
H	5.14719066	4.09483618	7.12181642
O	3.20170316	3.97558811	7.70497526
C	7.32472429	5.20665987	12.66205557
C	8.69467578	4.84399644	12.07967924
H	7.25307300	4.79145596	13.67662768
C	7.17313471	6.73604370	12.73019407
C	5.88425239	7.19352564	13.41086537
H	8.84681898	3.76561153	12.09632823
H	8.77905135	5.19034324	11.04898690
H	9.48211731	5.31377424	12.66883760
H	8.02906889	7.13859696	13.28110274
H	7.21055984	7.14197502	11.71347750
H	5.88371074	8.28036451	13.49561718
H	5.00764967	6.89491781	12.83627319

C	4.97604872	0.45076804	11.10238096
H	5.04557563	-0.62826664	10.91877659
H	5.19269915	0.98766601	10.17106807
O	3.66791039	0.74057724	11.54370590
C	3.88790338	1.56979595	15.90996846
C	4.24515566	0.63041092	17.06597088
H	2.85665988	1.91860653	16.05648626
C	4.82464573	2.78999504	15.89455957
C	4.17260299	4.00661533	15.24005663
H	3.64341388	-0.27687910	17.02238030
H	4.05240299	1.12932659	18.01547291
H	5.29917537	0.35440297	17.02806704
H	5.75362288	2.53125255	15.37443846
H	5.08695159	3.04769569	16.92546653
H	3.29680806	4.31912573	15.80988190
H	4.88023307	4.83523788	15.21141041
C	0.09854995	-1.02310496	15.71898435
H	-0.83290084	-1.60671418	15.65854849
H	0.93098730	-1.70369365	15.94221598
O	-0.00000000	0.00000000	16.68967114
C	0.96546459	-2.07086340	11.04250921
C	0.25768140	-1.35818216	9.87919029
N	1.02511515	-1.21565800	12.20610042
O	-0.08819343	-1.96463177	8.89721937
H	-1.07846553	-1.59041470	1.38130108
H	0.35834591	-2.94831547	11.30254935
H	-0.13321180	0.31647082	2.85179015
H	2.86235357	1.27583893	4.59272739
H	3.22147484	4.10840522	11.54066474
O	0.05891720	-0.05758546	9.98855382
S	0.00000000	0.00000000	0.00000000
C	-1.24499940	-0.52609960	1.20506049
C	-1.16935447	0.25076459	2.51260838
C	-1.74612898	1.64838353	2.33413576
H	-1.74661718	-0.26417808	3.28632142
O	-2.18862075	2.03567838	1.26424196
H	0.21654013	1.20349993	0.51548530
H	-2.23341332	-0.39997100	0.75470056
C	2.39452815	-2.50776410	10.64381149
C	3.09582380	-3.13546628	11.85323390
C	2.36638643	-3.49794550	9.47839239
H	2.95030205	-1.61142679	10.33736336
H	4.11777601	-3.40322713	11.58589560
H	3.12823188	-2.43746206	12.68840663
H	2.56965755	-4.03535092	12.17367466
H	1.75300566	-4.36431442	9.72797213

P	2.64222515	1.94949402	11.11021419
O	3.28418413	3.21574746	11.91235226
O	1.34341181	1.56078321	11.73112196
O	2.71261896	2.17423810	9.62716386
H	1.46810026	0.36568231	4.70391254

[3Mpa]RQASISI[pS]V (MHC3)

C	3.19080143	3.44779057	2.85646910
C	2.82397097	4.76624424	3.57014532
N	2.82649645	2.29286966	3.65673847
O	3.64098822	5.66412480	3.64482805
H	4.20117888	0.44628114	5.64579013
H	2.61777175	3.42410581	1.92258730
H	3.03335351	2.32211702	4.65219164
C	0.49862660	3.97953622	4.23684956
C	0.49952693	3.73482677	5.75766042
N	1.56885278	4.94084898	4.02395857
O	0.57394038	4.68370040	6.50882867
H	-3.01545232	1.23935095	1.67335560
H	0.67689253	3.07214114	3.66355915
H	1.45734549	5.78836580	4.57088043
H	-9.14504862	-3.51332454	4.18623956
C	0.33181705	2.06897725	7.53964132
C	0.51182251	0.56699733	7.74038620
N	0.49822359	2.43397437	6.13058162
O	-0.00000000	-0.00000000	8.67967553
H	1.28630850	2.31945269	9.44325001
H	-0.68604044	2.32319614	7.86513140
H	0.09245713	1.77094320	5.48052538
C	1.46374021	-1.50746179	6.98082928
C	0.11324697	-2.17197778	6.67235126
N	1.36251924	-0.05979895	6.88051054
O	-0.22867422	-3.18996427	7.22891657
H	-9.58219888	-2.48895317	1.23712499
H	1.70793964	-1.76389980	8.01924048
H	1.57510395	0.35170789	5.97934257
C	-2.05958432	-1.59082728	5.79986046
C	-2.49594291	-0.14344735	6.04265073
N	-0.60821357	-1.55571542	5.70243900
O	-1.81659070	0.80043095	5.65291662
H	-1.64639027	-5.38230140	4.16356147
H	-2.33533965	-2.23884178	6.63983865
H	-0.26225493	-0.66354328	5.36115753
C	-4.36793989	1.26170944	6.74897367
C	-4.98206223	1.54587757	5.37211630

N	-3.68339755	-0.01646189	6.64198148
O	-4.61448149	2.50498009	4.71123714
H	4.06851194	-1.88634341	7.27538306
H	-3.62609512	2.04734679	6.93361420
H	-4.20039808	-0.81126215	6.99787861
C	-6.36642521	0.71067807	3.56297875
C	-5.68711567	-0.41505657	2.75758764
N	-5.85816824	0.62828834	4.92418087
O	-4.85614730	-0.11195477	1.91639253
H	-10.27733516	0.13821231	1.08780293
H	-5.95317929	1.63083470	3.12826761
H	-6.30685199	-0.02346707	5.55853617
C	-6.95051826	-2.34552666	3.78906645
C	-7.53375605	-3.46439509	2.92146225
N	-5.89368631	-1.72371535	3.03813072
O	-6.94069239	-3.88486095	1.94500865
H	-6.68633023	-0.31514821	7.67694677
H	-7.72126546	-1.62030405	4.03991689
H	-5.46046625	-2.35027955	2.36247568
C	4.68931878	3.44445953	2.50595849
C	5.60884417	3.46249188	3.73588739
H	4.87942242	4.34498744	1.91650854
H	4.90856309	2.57513537	1.87941766
C	5.94468070	2.05615514	4.26800388
H	6.54021645	3.97078832	3.47283399
H	5.13901265	4.04553828	4.53129888
N	6.13676588	2.10628719	5.71195375
H	5.15012274	1.34470937	4.03788945
H	6.87075042	1.68810055	3.81561907
C	5.06183080	2.03615079	6.57111343
N	5.00729149	2.65702817	7.68918992
N	3.99283246	1.27298696	6.18541667
H	6.83256616	2.78552788	5.99586162
H	-10.99439855	-2.88151817	0.24342895
H	3.38227444	1.06551637	6.96293947
C	-0.83231047	4.61532067	3.80596410
H	-0.70483909	5.03061851	2.80224337
H	-1.07062782	5.43513602	4.48939046
C	-1.97267383	3.59790672	3.79741839
C	-1.86201296	2.62012428	2.63829735
O	-0.83089822	2.47457030	1.98421174
N	-2.98136708	1.94304187	2.38987410
H	-1.99157807	3.01734238	4.72489977
H	-2.92986365	4.11878798	3.72918543
H	-3.78416135	2.04790656	2.98754809
C	1.35087098	2.76132985	8.44937523

H	2.36391071	2.63200246	8.06774337
H	1.13635385	3.82471881	8.52341432
C	2.54817963	-2.04264574	6.03519881
H	2.51854462	-3.14235279	6.05508122
H	2.36067856	-1.69822055	5.01341382
O	3.82803959	-1.56773710	6.39704314
C	-2.76351943	-2.07491786	4.50968657
C	-2.14996185	-1.42956802	3.26784511
H	-3.80250759	-1.72261538	4.58814946
C	-2.83284782	-3.60805806	4.40345608
C	-1.48357585	-4.32171849	4.35678929
H	-1.12935365	-1.77928991	3.11701806
H	-2.13632341	-0.34574947	3.37988195
H	-2.74201591	-1.67753104	2.38729027
H	-3.39973600	-3.84734595	3.49686154
H	-3.40306069	-3.98883377	5.25741500
H	-0.96459278	-4.22372522	5.30837370
H	-0.84944384	-3.92183624	3.56528989
C	-5.38187770	1.17191345	7.92111794
H	-4.86426240	1.49423503	8.83443666
H	-6.21571253	1.86274281	7.73008074
O	-5.82033696	-0.13755439	8.11385736
C	-7.90330105	0.82518292	3.53501579
C	-8.28538094	2.21994663	4.04168113
H	-8.34590921	0.09935018	4.22310834
C	-8.42984778	0.56026853	2.11695145
C	-9.95091262	0.41531464	2.09034838
H	-7.85173249	2.38308733	5.02825904
H	-7.92075413	2.99440798	3.36533308
H	-9.36769823	2.30614085	4.12555626
H	-7.98294563	-0.36579583	1.73978296
H	-8.12120318	1.37191980	1.44947911
H	-10.26916139	-0.35979197	2.78857042
H	-10.43889581	1.35061427	2.36404515
C	-6.43897700	-2.95514983	5.12905834
H	-5.71466304	-2.24485639	5.55829549
H	-5.93517503	-3.91225111	4.94342837
O	-7.50380410	-3.16290687	5.98756596
C	-9.46076787	-4.89942975	2.61105152
C	-9.95988436	-6.01387325	3.52062451
N	-8.71103687	-3.92480300	3.36465099
O	-10.67077942	-6.90292694	3.13893072
H	1.45040954	-1.06270328	1.54439592
H	-8.78075353	-5.34894731	1.87438161
H	2.73974688	0.96086085	1.20566618
H	5.84496262	3.19260365	7.91082401

H	-9.73568367	-3.14748733	7.93678791
O	-9.53412841	-5.90717920	4.77457429
S	-0.00000000	0.00000000	0.00000000
C	0.89001289	-0.12787997	1.56423058
C	1.81935422	1.06484814	1.78533554
C	2.15247920	1.19760148	3.25658742
H	1.31580695	1.97615800	1.45671296
O	1.81015417	0.35460136	4.08199142
H	-0.68572443	1.08569489	0.34363867
H	0.17172494	-0.16834728	2.38419888
C	-10.65832352	-4.25952087	1.87055881
C	-11.59394602	-3.54529519	2.84877919
C	-10.15124266	-3.30414910	0.78963636
H	-11.20281642	-5.08468779	1.39356868
H	-11.96144408	-4.24369921	3.60048863
H	-12.44710237	-3.13570877	2.30828334
H	-11.08086703	-2.72826299	3.35665931
H	-9.50669103	-3.83125275	0.08618750
P	-8.65058028	-1.96628117	6.36171488
O	-9.75536360	-2.09853380	5.37141969
O	-7.90716270	-0.67648955	6.50068926
O	-9.07381060	-2.46858988	7.82460750
H	4.34161646	2.44344972	8.45623302

3) Ideal binding conformations

[3Mpa]RQASISISV (control)

C	0.55794243	-2.13439848	5.68233031
C	0.75530534	-3.65384348	5.79789144
N	0.61189564	-1.79353666	4.26423449
O	1.85534600	-4.16711052	5.78809840
H	3.51410578	-0.61981044	10.94451618
H	-0.43487151	-1.85082212	6.04817130
H	1.52446821	-1.80777228	3.83380548
C	-0.35588769	-5.78760633	6.12764609
C	0.45648339	-6.16051799	7.37638435
N	-0.39456274	-4.34315617	5.95413758
O	0.88639298	-7.29103293	7.53221462
H	0.49137810	-9.22873134	4.40657453
H	0.16150800	-6.24382538	5.27174092
H	-1.27334128	-3.88495731	5.76236384
H	-0.25243976	-2.10183948	20.08355785
C	1.68713568	-5.29374494	9.26975312
C	2.17653475	-3.89672617	9.62468830
N	0.60558971	-5.18067938	8.28981112
O	3.36155748	-3.62671050	9.65763209
H	0.41501181	-5.65712698	11.00793534
H	2.52525282	-5.81519994	8.78787725
H	0.34186859	-4.24412067	8.01172417
C	-0.12771782	-2.98396504	10.23337910
C	-0.29113134	-2.64173257	11.72373670
N	1.26500128	-2.91258659	9.84784182
O	-1.35141338	-2.24170315	12.16261874
H	-2.09379005	-3.21081101	8.25695267
H	-0.50769514	-4.00108207	10.09233356
H	1.72812651	-2.02976735	10.07662200
C	0.77474865	-2.83341290	13.89847646
C	1.19824795	-4.23800081	14.32300919
N	0.82688793	-2.81443392	12.44756111
O	1.57509131	-5.08202165	13.52670302
H	-0.91260186	-0.56975918	13.93025788
H	-0.26477589	-2.66638048	14.20505216
H	1.58595236	-3.35000426	12.04526700
C	1.48009839	-5.73238933	16.23133031
C	1.59432960	-5.44891778	17.73006461
N	1.09792778	-4.45896452	15.64332296
O	1.95896751	-4.35787001	18.12794459
H	1.45761110	-8.47546630	15.80660432
H	2.46096956	-6.03243790	15.83638553

H	0.82110121	-3.72084469	16.27850522
C	1.00066632	-6.05864040	19.97496839
C	0.01162523	-4.88339776	20.03163654
N	1.20710929	-6.42516482	18.57659299
O	0.04369338	-4.04443691	20.91326571
H	0.09646965	-8.87256153	23.10628506
H	1.94926159	-5.69552161	20.39150038
H	0.84359810	-7.29933359	18.22736187
C	-1.71167690	-3.70437254	18.76273842
C	-0.77903955	-2.61950729	18.18689587
N	-0.92059443	-4.89255653	19.05595926
O	-0.61759459	-2.48652561	16.97547954
H	-1.58762664	-4.02797052	16.18062429
H	-2.18020197	-3.34955450	19.68776078
H	-0.80803193	-5.51065196	18.26290766
C	1.64840344	-1.43065896	6.48395957
C	1.58887256	0.09416880	6.36080281
H	1.50656895	-1.71104358	7.53269157
H	2.62953510	-1.80064057	6.17063683
C	2.30689622	0.75655349	7.54107070
H	0.54694421	0.42903585	6.35064248
H	2.05509079	0.41423803	5.42469318
N	1.48656912	0.61553193	8.74813304
H	3.28318383	0.29077803	7.69781037
H	2.46220805	1.82333548	7.33356600
C	2.06837880	0.40072906	9.99883688
N	1.67100257	0.96712414	11.06333273
N	2.98858279	-0.61669071	10.08744765
H	0.79344928	1.35420246	8.79838008
H	3.67290176	-2.24234600	19.80750214
H	3.49592965	-0.90402694	9.27010664
C	-1.79462898	-6.31378359	6.21854769
H	-2.28692927	-5.85841867	7.08437245
H	-2.33417226	-6.00312969	5.31853683
C	-1.86084059	-7.84220215	6.33620280
C	-1.25827071	-8.48682648	5.09012575
O	-1.89078696	-8.62656302	4.06513276
N	0.02677260	-8.87283919	5.22165866
H	-2.91113473	-8.13625226	6.39051153
H	-1.34908951	-8.18138209	7.23804278
H	0.56108575	-8.64169541	6.04275258
C	1.26737906	-6.10325887	10.49835783
H	2.10030351	-6.15279070	11.19800341
H	1.00354250	-7.11181040	10.18318594
C	-0.97104446	-2.03071762	9.36359856
H	-0.38454598	-1.13710791	9.12081382

H	-1.87062568	-1.73354894	9.91957994
O	-1.31718739	-2.65188461	8.13981637
C	1.67512552	-1.74425508	14.51407547
C	3.14422607	-1.98654197	14.15496694
H	1.56259598	-1.81724934	15.60363142
C	1.24170289	-0.33841981	14.07233097
C	-0.18400207	0.00897860	14.49748479
H	3.46476374	-2.97170008	14.49430880
H	3.29024278	-1.92500542	13.07548558
H	3.77187864	-1.23384722	14.63176728
H	1.93399406	0.38080314	14.52305608
H	1.33455069	-0.24645853	12.98427651
H	-0.37106824	1.06753449	14.31526488
H	-0.32934702	-0.19269034	15.56043152
C	0.43170000	-6.79565190	15.84276062
H	0.35066815	-6.82288629	14.74842412
H	-0.54245912	-6.52470551	16.26547834
O	0.77726204	-8.06675387	16.35490280
C	0.49760876	-7.27633644	20.77292574
C	1.51617984	-8.41830493	20.67256934
H	-0.45272298	-7.60541404	20.32841822
C	0.25778874	-6.88119029	22.24059528
C	-0.49230095	-7.95897546	23.02222859
H	1.14458657	-9.30056269	21.19189757
H	2.46476080	-8.12158347	21.12195942
H	1.69548496	-8.68966724	19.63258520
H	1.22010339	-6.67886478	22.72216943
H	-0.32770706	-5.95916663	22.27538714
H	-0.70508647	-7.59594280	24.02783898
H	-1.43788754	-8.19749545	22.53300883
C	-2.75966781	-4.11857673	17.71624110
H	-3.43660339	-4.86204824	18.15138594
H	-3.34274513	-3.23627314	17.41274663
O	-2.12927705	-4.71625365	16.60948464
C	0.70227136	-0.77392668	18.73114549
C	0.60171684	0.35441930	19.73882931
N	-0.20602702	-1.84436483	19.10541891
O	1.03086149	1.46046024	19.55622704
H	0.40477118	0.14393793	2.31393724
H	0.38680874	-0.39191892	17.75215181
H	0.66417636	-2.27996896	1.69558318
H	0.94736531	1.66649579	10.90865674
H	2.70659734	-1.05390409	20.70017886
O	0.00000000	0.00000000	20.87942927
S	0.00000000	0.00000000	0.00000000
C	-0.36003247	-0.37795154	1.73484745

C	-0.29736038	-1.88118033	2.02657035
C	-0.48101300	-2.10759311	3.51602558
H	-1.09860056	-2.39830523	1.49558795
O	-1.53242283	-2.46432016	4.01123770
H	-1.05877889	-0.61891099	-0.51399152
H	-1.33843479	0.01514935	2.01932368
C	2.15830066	-1.29099748	18.60933999
C	3.07474092	-0.19807681	18.05877122
C	2.67024367	-1.83741676	19.94211473
H	2.12713114	-2.11388223	17.88622838
H	2.67631505	0.19664121	17.12379198
H	4.06216442	-0.61722374	17.86560622
H	3.17818720	0.62425229	18.76592605
H	2.02224820	-2.63954613	20.29367454
H	2.33731044	1.09255889	11.85766025

[3Mpa]RQA[pS]ISISV (MHC1)

Same as low energy conformation coordinates

[3Mpa]RQASI[pS]ISV (MHC2)

C	-0.78703818	-3.19000406	2.65415710
C	-2.11212217	-2.42003336	2.81570642
N	0.19861768	-2.36941998	1.96868027
O	-2.96260284	-2.38869788	1.93739361
H	-5.18409990	-3.32331820	1.78481234
H	-0.38491130	-3.41028535	3.64967588
H	0.15991502	-2.32569103	0.96071306
C	-3.37076880	-1.03941940	4.43101314
C	-3.62232425	0.07684966	3.40455037
N	-2.21929280	-1.81830184	4.00761562
O	-4.64685302	0.14274845	2.75860352
H	-2.07450160	-3.51685960	6.34040474
H	-3.09964463	-0.58968758	5.39136319
H	-1.47394327	-1.89445875	4.69082102
H	2.55014991	-2.04283355	11.30291112
C	-1.53409384	1.24408157	4.17809443
C	-0.45901089	1.95210117	3.36286931
N	-2.62022891	0.96604967	3.25257239
O	-0.68008542	2.32559373	2.21980117
H	-2.40728600	3.09653184	4.88057056
H	-1.14115382	0.30974076	4.58847907
H	-2.80735287	1.71240684	2.59265946
C	1.37838925	1.50409492	5.02631509
C	2.70651103	1.03977553	4.41410672

N	0.72119171	2.24121391	3.95583129
O	3.10314750	1.52679546	3.36246085
H	2.37240313	1.29988949	12.40576173
H	0.76505153	0.65111240	5.32439078
H	1.39591488	2.63374466	3.30128867
C	3.17117397	-0.66749652	6.23024447
C	3.70111125	-2.06277508	5.90143361
N	3.45244930	0.12570538	5.04889561
O	4.81198306	-2.20195321	5.42448214
H	6.99040689	1.50957565	7.88435021
H	2.09896221	-0.67201531	6.42324444
H	4.29428391	-0.15013274	4.55629249
C	1.50391827	-3.25297788	6.31884446
C	1.09937937	-4.68451910	5.98383567
N	2.95969617	-3.16149254	6.21414241
O	0.26344333	-4.91291086	5.12472253
H	0.68913166	3.82944152	5.24047414
H	1.03403055	-2.59336340	5.57959503
H	3.40276319	-4.00381357	5.86845026
C	2.32710950	-5.69064025	7.94747129
C	1.19667595	-6.03633398	8.93003259
N	1.71331609	-5.71256598	6.62310917
O	0.32219484	-6.80663782	8.58703671
H	5.84734658	-5.45516436	8.79259246
H	2.75690543	-4.70649983	8.13221869
H	1.23641823	-6.59096783	6.44128807
C	1.93697085	-4.40795606	10.72982808
C	0.97167983	-3.21536871	10.79151885
N	1.21509156	-5.54370235	10.19393064
O	-0.22876951	-3.35998972	10.65762127
H	2.66804932	-3.05397632	13.17603758
H	2.81695977	-4.17526746	10.12553775
H	0.33407578	-5.71268328	10.66902995
C	-0.98254959	-4.50031186	1.88273869
C	-1.22751926	-4.29596626	0.37733089
H	-1.82641245	-5.03155015	2.33632861
H	-0.09480063	-5.12160853	2.02762699
C	-2.39616528	-5.13603052	-0.16040389
H	-1.46035601	-3.24887806	0.16391968
H	-0.32917981	-4.56308615	-0.18594448
N	-3.64569561	-4.59177442	0.32814160
H	-2.32267289	-6.17801926	0.16102447
H	-2.40483791	-5.08903161	-1.25361910
C	-4.16030206	-5.03437374	1.50257410
N	-3.74090644	-6.16512873	1.99832883
N	-5.15425201	-4.29809710	2.03971140

H	-3.79117354	-3.60769588	0.13456507
H	3.32952105	0.12789346	11.48845983
H	-5.37137611	-4.51483616	3.00376285
C	-4.63200115	-1.88535779	4.61376241
H	-5.45227036	-1.19660397	4.83238788
H	-4.87083759	-2.39743333	3.68010884
C	-4.49761156	-2.89544568	5.76238923
C	-3.73265586	-4.13259278	5.31986780
O	-4.24542534	-4.95937170	4.57041874
N	-2.49554035	-4.25725430	5.79997524
H	-4.01640274	-2.42727460	6.62305471
H	-5.49953816	-3.22351524	6.04997901
H	-1.91465927	-5.01604363	5.48379737
C	-2.02953031	2.16487565	5.30071678
H	-2.82913305	1.67404834	5.85243799
H	-1.21597138	2.38225350	5.98830046
C	1.62873391	2.44728907	6.24181830
H	2.69760713	2.71808615	6.27718211
H	1.36568279	1.91740290	7.16358416
O	0.83499326	3.60098722	6.17154632
C	3.92975408	-0.09465026	7.46453102
C	3.97028455	-1.12935316	8.59086177
H	3.35666717	0.77341009	7.81663879
C	5.34283046	0.37743951	7.08700694
C	6.05954270	1.07128359	8.24497052
H	2.96348118	-1.46825286	8.82220381
H	4.57770218	-1.99020132	8.31097901
H	4.39331463	-0.68863740	9.49233847
H	5.93569128	-0.47469413	6.74197985
H	5.26357834	1.08891604	6.25961541
H	6.30138347	0.36796959	9.04185096
H	5.43925749	1.86835845	8.65842129
C	0.96419813	-2.88639148	7.71834698
H	1.71038502	-3.15376346	8.47063372
H	0.05213418	-3.46833229	7.90547532
O	0.71350705	-1.51874330	7.83162721
C	3.45966948	-6.73877760	8.04600754
C	2.98933968	-8.13674242	7.63370349
H	3.76480314	-6.77057401	9.10126066
C	4.66583985	-6.31027073	7.19140987
C	5.46371980	-5.16535853	7.81350131
H	2.07162886	-8.40659694	8.15410551
H	2.80872721	-8.18316081	6.55937470
H	3.75864897	-8.86687471	7.88390899
H	4.31450889	-6.02145554	6.19471543
H	5.32851535	-7.17265445	7.07042949

H	6.30534082	-4.91204620	7.16944053
H	4.84868233	-4.27325497	7.93368825
C	2.42227160	-4.73870623	12.16851269
H	3.04430458	-5.64111270	12.11719542
H	1.55548843	-4.94313492	12.81309608
O	3.20450410	-3.68945196	12.66244324
C	0.79406187	-0.88127057	11.42215213
C	0.84585796	-0.84318762	12.94777309
N	1.58365504	-2.03251234	11.00621635
O	1.59147192	-1.54014357	13.58603269
H	2.28837786	0.61787681	0.17776272
H	-0.23891450	-1.06701034	11.10788418
H	2.90064333	-0.56828636	2.28603990
H	-4.12476866	-6.32897035	2.93232126
H	-1.52128094	-1.97926680	9.53530836
O	0.00000000	0.00000000	13.53450081
S	0.00000000	0.00000000	0.00000000
C	1.53247391	0.33450611	0.91223476
C	2.04602344	-0.88475782	1.68575296
C	1.00626188	-1.50164760	2.60984181
H	2.37923627	-1.64973017	0.97943511
O	0.93530253	-1.27814119	3.80834483
H	-0.79646713	0.72088659	0.77927493
H	1.38064211	1.17281275	1.58858512
C	1.31181460	0.40266152	10.73555431
C	0.23736239	1.48895158	10.67942936
C	2.58663131	0.92152263	11.40540821
H	1.54328312	0.11580343	9.70282780
H	-0.02248915	1.85202120	11.67628354
H	-0.65797018	1.10870303	10.19081556
H	0.60994090	2.33242476	10.09853737
H	3.01081718	1.73096655	10.81176376
P	-0.82915973	-0.85381544	7.70086829
O	-1.41613061	-1.08186547	9.20694901
O	-1.58820263	-1.70220227	6.72333410
O	-0.62937868	0.58796087	7.48772849
H	-3.17207187	-6.96466999	1.66014005

[3Mpa]RQASISI[pS]V (MHC3)

C	-2.04621320	-2.16881825	3.83886508
C	-2.00874041	-3.61754496	4.33842061
N	-1.73381667	-2.03184257	2.42810025
O	-1.76926122	-3.83467922	5.52463430
H	2.80756818	-2.98730938	4.39255939
H	-3.07379091	-1.79763728	3.96926812

H	-1.06829816	-2.66321495	2.01517472
C	-2.50576775	-4.73651110	2.10838150
C	-1.27836599	-4.69601366	1.19169409
N	-2.22258764	-4.66384209	3.52450713
O	-1.45555913	-4.45692714	0.00339696
H	-7.04468805	-4.70513828	0.24462785
H	-3.13342067	-3.88636077	1.82153825
H	-2.26520758	-5.55193903	4.01961068
H	0.52466617	-0.82224159	12.87894155
C	0.56976445	-5.10124397	2.93591456
C	0.14652690	-6.45715487	3.52124547
N	-0.01639692	-4.90145847	1.62008477
O	0.21789640	-7.48250439	2.87469767
H	2.43377333	-4.20608412	2.33072206
H	0.29196943	-4.27466138	3.59770952
H	0.63987386	-4.92804600	0.85134089
C	-0.66238433	-7.58918522	5.52256647
C	-1.06084169	-7.17767421	6.94207367
N	-0.22800520	-6.39412251	4.81146810
O	-2.16704513	-7.46957747	7.36557166
H	-0.50402601	0.89314388	10.23747932
H	0.15623095	-8.32067263	5.53513143
H	-0.39369565	-5.49861430	5.25218984
C	1.17150082	-6.07909933	7.41622887
C	1.18177374	-4.60065296	6.98715703
N	-0.20185282	-6.46364796	7.69252639
O	1.41810935	-4.29614845	5.82271894
H	4.53474774	-6.94813109	9.93328654
H	1.53554360	-6.65665856	6.55881023
H	-0.60222692	-6.17248314	8.58098801
C	0.86792092	-2.30421219	7.55981988
C	-0.11553584	-1.40662799	8.29010125
N	0.82964452	-3.70452535	7.92778645
O	0.05111058	-0.19476042	8.24676888
H	-3.23175742	-7.11835047	5.68732743
H	0.63320443	-2.25258368	6.49089572
H	0.99796400	-3.91020951	8.91132116
C	-1.87928383	-3.13483107	8.85974329
C	-1.69710411	-4.15066962	9.98318199
N	-1.13165490	-1.90602078	9.03295618
O	-1.86711379	-5.33623360	9.76638348
H	-3.12198815	-5.26807342	7.50851867
H	-1.58569790	-3.61413235	7.92046931
H	-1.64599480	-1.17623488	9.51708068
C	-0.56422231	-2.71955834	11.68299054
C	-1.23600674	-1.37714353	12.00411791

N	-1.50369552	-3.74056206	11.27055184
O	-2.36373521	-1.10699786	11.62719737
H	1.77973287	0.11493463	7.37490057
H	0.08899791	-2.49024266	10.83322613
H	-1.53647855	-4.53973248	11.89486769
C	-1.10900767	-1.36149167	4.75075715
C	-0.96723626	0.12705412	4.43169141
H	-1.50420732	-1.45764739	5.76348843
H	-0.12972535	-1.84425215	4.73920250
C	0.21243803	0.46414309	3.50186790
H	-0.81558925	0.66076175	5.37487681
H	-1.88063783	0.50791833	3.96769704
N	1.45013081	-0.13862359	3.95901833
H	0.00548122	0.13502563	2.47809929
H	0.35363658	1.54943223	3.49154451
C	1.80313612	-1.41784509	3.63544824
N	1.36311500	-2.07337969	2.60193380
N	2.73422092	-1.98481403	4.42750142
H	1.77448020	0.17325481	4.86793356
H	-1.81925494	1.96948277	10.73689421
H	2.93812673	-1.54058648	5.30691419
C	-3.28728469	-6.03761629	1.83791662
H	-4.03449318	-6.16144306	2.62681230
H	-2.60377324	-6.88978023	1.88860207
C	-3.97944883	-6.02563514	0.46707459
C	-5.04477880	-4.94401903	0.40238707
O	-4.79116885	-3.75580567	0.44770280
N	-6.31564441	-5.39177256	0.30829392
H	-4.41493468	-7.00943382	0.27977787
H	-3.24236532	-5.81434053	-0.31141117
H	-6.54152830	-6.36443606	0.24075200
C	2.08929136	-5.13858870	2.77707108
H	2.38115635	-5.97654059	2.14427307
H	2.54600069	-5.25677653	3.75890834
C	-1.89307073	-8.21320849	4.82806959
H	-1.63593021	-8.49481582	3.80384496
H	-2.18866073	-9.11263441	5.38710181
O	-2.95577599	-7.28877447	4.76782681
C	2.05784581	-6.35894935	8.64751784
C	2.00961622	-7.85394286	8.97388073
H	1.66071514	-5.80047130	9.50429117
C	3.48805044	-5.87548311	8.35821863
C	4.38707689	-5.92318931	9.59230469
H	2.56083403	-8.05711767	9.89057774
H	2.44680721	-8.44026331	8.16377309
H	0.97703881	-8.17243228	9.11698918

H	3.43870100	-4.84021853	8.00444859
H	3.92269154	-6.48066422	7.55419620
H	5.36083677	-5.49811111	9.34803204
H	3.94791186	-5.34168564	10.40173892
C	2.28798281	-1.70290714	7.73742609
H	3.02715782	-2.42133859	7.36952180
H	2.48680162	-1.48187513	8.79178068
O	2.38611500	-0.52731484	6.95718512
C	-3.38812003	-2.75209396	8.83793747
C	-3.65680908	-1.76642223	7.69575933
H	-3.59366426	-2.24291527	9.79032242
C	-4.32791149	-3.96499103	8.74814976
C	-4.12283550	-4.84154671	7.51362041
H	-4.71404246	-1.50212265	7.68473979
H	-3.39796883	-2.20996171	6.73507057
H	-3.07659547	-0.85348233	7.82578763
H	-5.35366339	-3.58094787	8.75141065
H	-4.21083936	-4.58035416	9.64395045
H	-4.26681469	-4.27458282	6.59415366
H	-4.84275659	-5.66008992	7.53090125
C	0.33025442	-3.26717183	12.81440897
H	-0.13239613	-3.09023205	13.79423188
H	0.45495933	-4.35038514	12.66992261
O	1.56257792	-2.61929013	12.77889134
C	-0.83234072	0.79961055	13.01234191
C	-0.51790607	1.05814774	14.47624497
N	-0.41609313	-0.53545975	12.63928726
O	-0.70746254	2.10997789	15.02242848
H	-0.44744200	-2.31060985	-0.08714523
H	-1.92204578	0.85699943	12.88801468
H	-2.99870992	-0.61744729	-0.30755560
H	0.77550638	-1.50125053	1.99764788
H	4.50510547	-2.69533668	12.53927469
O	0.00000000	-0.00000000	15.10444903
S	0.00000000	0.00000000	0.00000000
C	-1.00827506	-1.44289875	-0.43393418
C	-2.41084416	-1.41176083	0.15852708
C	-2.49795224	-1.20418461	1.65994299
H	-2.90056587	-2.36769540	-0.06205178
O	-3.23559752	-0.37727143	2.15433787
H	-0.93809809	0.91992079	-0.19741485
H	-1.08892189	-1.50875601	-1.52124185
C	-0.16501113	1.88389572	12.13414568
C	1.35715875	1.72522293	12.11662016
C	-0.73650626	1.84312145	10.71708541
H	-0.42008997	2.84690069	12.59651789

H	1.80470280	2.56133839	11.57965412
H	1.75139027	1.71359930	13.13304917
H	1.64872878	0.80121573	11.61647132
H	-0.30134410	2.64476620	10.12054000
P	2.57180463	-2.63755502	11.40843001
O	2.75725038	-1.22070898	11.01411558
O	2.01684922	-3.64312087	10.45044329
O	3.92160838	-3.26776321	12.04483241
H	1.07488853	-2.96228540	2.40094884
